# Anhedonia and general distress show dissociable ventromedial prefrontal cortex connectivity in major depressive disorder

**DOI:** 10.1038/tp.2016.80

**Published:** 2016-05-17

**Authors:** C B Young, T Chen, R Nusslock, J Keller, A F Schatzberg, V Menon

**Affiliations:** 1 Department of Psychiatry and Behavioral Sciences, Stanford University School of Medicine, Stanford, CA, USA; 2 Department of Psychology, Northwestern University, Evanston, IL, USA; 3 Department of Neurology and Neurological Sciences, Stanford University School of Medicine, Stanford, CA, USA; 4 Stanford Neuroscience Institute, Stanford University School of Medicine, Stanford, CA, USA

## Abstract

Anhedonia, the reduced ability to experience pleasure in response to otherwise rewarding stimuli, is a core symptom of major depressive disorder (MDD). Although the posterior ventromedial prefrontal cortex (pVMPFC) and its functional connections have been consistently implicated in MDD, their roles in anhedonia remain poorly understood. Furthermore, it is unknown whether anhedonia is primarily associated with intrinsic ‘resting-state' pVMPFC functional connectivity or an inability to modulate connectivity in a context-specific manner. To address these gaps, a pVMPFC region of interest was first identified using activation likelihood estimation meta-analysis. pVMPFC connectivity was then examined in relation to anhedonia and general distress symptoms of depression, using both resting-state and task-based functional magnetic resonance imaging involving pleasant music, in current MDD and healthy control groups. In MDD, pVMPFC connectivity was negatively correlated with anhedonia but not general distress during music listening in key reward- and emotion-processing regions, including nucleus accumbens, ventral tegmental area/substantia nigra, orbitofrontal cortex and insula, as well as fronto-temporal regions involved in tracking complex sound sequences, including middle temporal gyrus and inferior frontal gyrus. No such dissociations were observed in the healthy controls, and resting-state pVMPFC connectivity did not dissociate anhedonia from general distress in either group. Our findings demonstrate that anhedonia in MDD is associated with context-specific deficits in pVMPFC connectivity with the mesolimbic reward system when encountering pleasurable stimuli, rather than a static deficit in intrinsic resting-state connectivity. Critically, identification of functional circuits associated with anhedonia better characterizes MDD heterogeneity and may help track of one of its core symptoms.

## Introduction

Little is known about the neural underpinnings of individual symptoms of major depressive disorder (MDD), such as anhedonia, as the disorder has been primarily examined as a unitary construct. However, the research domain criteria framework highlights the growing recognition that complex psychiatric disorders such as MDD need to be more fully characterized by identifying potentially distinct neurobiological mechanisms associated with individual symptom clusters.^1^ Examining the relationships between specific symptoms and brain circuits, such as anhedonia and mesolimbic and cortical pathways involved in reward processing, has important implications for understanding the etiology of MDD symptoms and developing targeted treatments.^1, 2^ Here, we take a research domain criteria-like approach^1^ to distinguish symptom features and investigate the functional brain circuits implicated in MDD and reward processing. Our primary goal was to investigate the specificity of neurofunctional pathways associated with anhedonia by conducting differential circuit analysis with task-based and resting-state functional magnetic resonance imaging (fMRI) in individuals with MDD. Our second goal was to examine whether connectivity patterns related to anhedonia are specific to distinct anatomical ventromedial prefrontal cortex (VMPFC) subregions. A third goal of this study was to investigate whether anhedonia-specific pathways observed in MDD are also present in healthy controls.

A clinical diagnosis of MDD requires the presence of at least one of two symptoms: depressed mood and anhedonia, defined as diminished interest or pleasure in response to rewarding stimuli.^3^ Recent estimates suggest that approximately 37% of individuals with MDD experience clinically significant anhedonia.^4^ Anhedonia involves specific impairments in motivation and reward-based decision-making,^5, 6^ and is linked to abnormal activity in the brain regions important for reward processing.^7, 8, 9^ Anhedonia is also a predictor of poor treatment response in MDD,^10^ and is especially difficult to treat both pharmacologically and psychosocially.^11, 12, 13, 14, 15, 16, 17, 18, 19^ Given the significance of anhedonia in MDD and its relationship with reward-processing deficits, it is critical to identify the brain regions and functional circuits that are specifically associated with this symptom in affected individuals.

There have been relatively few neuroimaging studies examining anhedonia in MDD and fewer still have disentangled anhedonia from depression severity. Emerging evidence suggests that anhedonia is characterized by reduced activity in subcortical and ventromedial prefrontal cortex regions involved in reward processing and monitoring.^7, 8, 20, 21, 22^ Furthermore, the posterior VMPFC (pVMPFC) has been consistenty implicated in MDD in previous neuroimaging studies,^23, 24, 25, 26, 27^ as well as findings from psychopharmacology,^24, 28^ psychotherapy^29^ and deep-brain stimulation treatments.^30, 31^ Indeed, the pVMPFC is thought to be central to the pathophysiology of depression.^18, 32^ However, the unique effects of anhedonia on the pVMPFC circuits are currently unknown.

Understanding the pathophysiology of psychiatric disorders such as MDD requires better characterization of underlying task-modulated and intrinsic resting-state functional circuits. A network of brain regions that includes the pVMPFC, specifically its BA25/32pl subdivision, is particularly relevant to MDD. Anatomical tracing studies have identified pVMPFC projections to regions in the mesolimbic reward system, including the nucleus accumbens (NAc).^33, 34^ In addition, the pVMPFC has connections to limbic structures that are central to emotion-processing and hypothalamic regions that modulate autonomic reactivity.^33^ The animal models of depression have shown that optogenetic stimulation of medial prefrontal cortex cells that terminate in the NAc elicits antidepressant effects in mice,^35^ and these tracks are implicated in appetitive conditioning and reward-related hedonic behavior.^36^ To date, however, no study has examined the functional connectivity of this network in humans or determined whether its dysfunction is related to individual MDD symptoms. Furthermore, a critical question that has not yet been addressed is whether specific clinical symptoms are associated with intrinsic resting-state brain connectivity or an inability to modulate brain responses in a context-specific manner.

Previous studies of brain networks in MDD have separately focused on either task-modulated connectivity or resting-state connectivity.^8, 37, 38, 39, 40, 41^ Thus, it is unknown whether anhedonia in MDD is related to aberrant intrinsic (resting-state) functional circuits or an inability to engage reward- and emotion-related networks in a contextually appropriate manner. It is also unknown whether such deficits are specific to anhedonia, rather than general depressive symptoms. Here, we investigate the relationship of anhedonia with task-modulated and resting-state pVMPFC connectivity in current MDD patients and healthy controls. To assess the specificity of these relationships with anhedonia, we also examined general distress, which is common across anxiety and mood disorders and is associated with elevated negative affect, poor outcome and abnormalities in threat-related neural circuitry.^42, 43^ Given the anatomical connections of pVMPFC to reward-related regions and the close relationship of anhedonia to reward processing, we hypothesized that weakened pVMPFC connectivity with reward-related brain regions would be related to anhedonia but not general distress. By assessing both anhedonia and general distress, as well as including task- and resting-state fMRI, we are able to characterize functional brain circuits uniquely associated with anhedonia.

We first identified a pVMPFC region of interest (ROI) by performing a meta-analysis based on voxels identified in a recent review of mood and anxiety disorders.^32^ Critically, this allowed for an unbiased and theoretically motivated selection of the pVMPFC region most frequently implicated in mood and anxiety disorders. Our meta-analysis also identified a perigenual VMPFC region, which was used to examine the anatomical specificity of our pVMPFC findings. We used task-based fMRI with pleasant music and control stimuli because music is an ecologically relevant stimulus that is capable of evoking strong emotions^44, 45^ and feelings of pleasure.^46, 47^ Music reliably activates the brain's reward circuits,^48, 49, 50, 51, 52, 53, 54^ as well as superior temporal sulcus and auditory regions^45^ that send auditory and polymodal outputs to the VMPFC.^55, 56^ A passive music-listening task also facilitates a more direct comparison of pVMPFC connectivity differences between task and rest states without the potentially confounding effects of performance, decision-making and action. In summary, we used both resting-state and task-based fMRI to probe pVMPFC, and secondarily perigenual VMPFC, connectivity in relation to anhedonia and general distress symptoms of depression in MDD patients. We also investigated whether similar dissociations between anhedonia and general distress are seen in healthy controls despite constricted range on clinical measures.

## Materials and methods

### Participants

Twenty-five participants with current MDD and 25 healthy controls were recruited through community advertisements and were paid $75 for their participation. Twenty-one patients (M=46.10 years, s.d.=14.55; 11 females/10 males) and 22 healthy controls (M=34.55 years, s.d.=10.23; 14 females/8 males) were included in the final analyses after excluding participants for outlier data and excessive motion (>3 mm) during the fMRI scan. The resting-state connectivity analyses included 17 patients as three patients did not complete the resting-state scan and one patient had outlier data, as well as 16 healthy controls as three participants did not complete the resting-state scan and three participants had excessive motion during the scan. The depressed and control groups did not significantly differ in gender, *χ*^2^(1, *N*=42)=0.56, *P*>0.45, but did significantly differ in age, *t*(41)=3.02, *P*=0.004 (Supplementary Figure S1). Thus, grand mean-centered age was included as a covariate of no interest in all the analyses. The Stanford University School of Medicine Human Subjects Committee approved the protocol.

The exclusion criteria for both the groups included history of seizures, major medical illnesses, head trauma, neurological illnesses, pregnancy, current or recent (last 6 months) substance abuse, current or recent smoking habits and any metal in the body that precluded MRI. All the participants completed the Structured Clinical Interview for the DSM-IV,^57^ except for two patients who completed the Mini International Neuropsychiatric Interview (MINI);^58^ one patient completed the Mini International Neuropsychiatric Interview outside of the 30-day time window, but a current major depressive episode was confirmed at the time of testing using the mood module only. All the participants also completed the 24-item Hamilton Rating Scale for Depression (HDRS).^59^ All the patients met DSM-IV criteria for current major depressive episode and all the healthy controls were free of Axis I or Axis II disorders. The eligible participants completed the scan within 30 days of the interview and the majority completed the scan within 2 weeks. Before scanning, all the participants were re-interviewed using the HDRS^59^ and completed the Mood and Anxiety Symptom Questionnaire (MASQ) 62-item Short Form.^60, 61^

### Measuring anhedonia and general distress

The MASQ consists of four scales: (1) general distress depressive symptoms (MASQ-GDD), which measures overall negative affect related to depression, (2) anhedonic depression (MASQ-AD), which assesses high and low positive affect,^60, 61, 62, 63^ (3) general distress anxious symptoms, which assesses negative affect related to anxiety and (4) anxious arousal, which is specific to anxiety.^60, 61^ The MASQ-AD can be further decomposed into a reverse-scored 14-item high positive affect factor, assessing anhedonia and an eight-item depressive/low positive affect factor.^62, 63, 64^ To assess anhedonia independently of negative emotionality, we used the high positive affect factor of the MASQ-AD (MASQ-AD-PA) and the MASQ-GDD to probe anhedonia and depression symptoms, respectively. Supplementary Table S1 displays the correlations between scales and internal consistency of items in each scale.

### fMRI music-listening task

The participants listened and responded with a button press at the start and end of each music epoch. Stimuli were chosen on the basis of a previously published study^48^ and consisted of three classical musical pieces that were likely familiar and three that were likely unfamiliar (stimuli available at http://www.scsnl.stanford.edu). The Music and Scrambled stimuli were each presented for 22 to 28 s, followed by a 22-s rest epoch. The Scrambled pieces controlled for low-level acoustic features and attention to complex auditory events, and thus served as the control for the Music pieces. Additional details are available in Supplementary Materials.

### Resting-state fMRI

For the resting-state fMRI scan, the participants were instructed to keep their eyes closed and remain still for the duration of an 8-min scan (one patient completed a 9-min 4-s scan).

### Postscan questionnaire

Following the fMRI session, the participants listened to the music epochs again and rated the Music and Scrambled pieces on a nine-point Likert scale (−4 to +4) on 10 different bipolar semantic differentials: exciting–calm, unpleasant–pleasant, tense–relaxed, annoying–unannoying, dissonant–consonant, angry–peaceful, happy–sad, moving–unmoving, boring–interesting and unfamiliar–familiar (Supplementary Table S2). The ratings for both the pieces were available for 18 of 21 patients and 19 of 22 healthy controls.

### fMRI data acquisition and analyses

**Figure 1:**
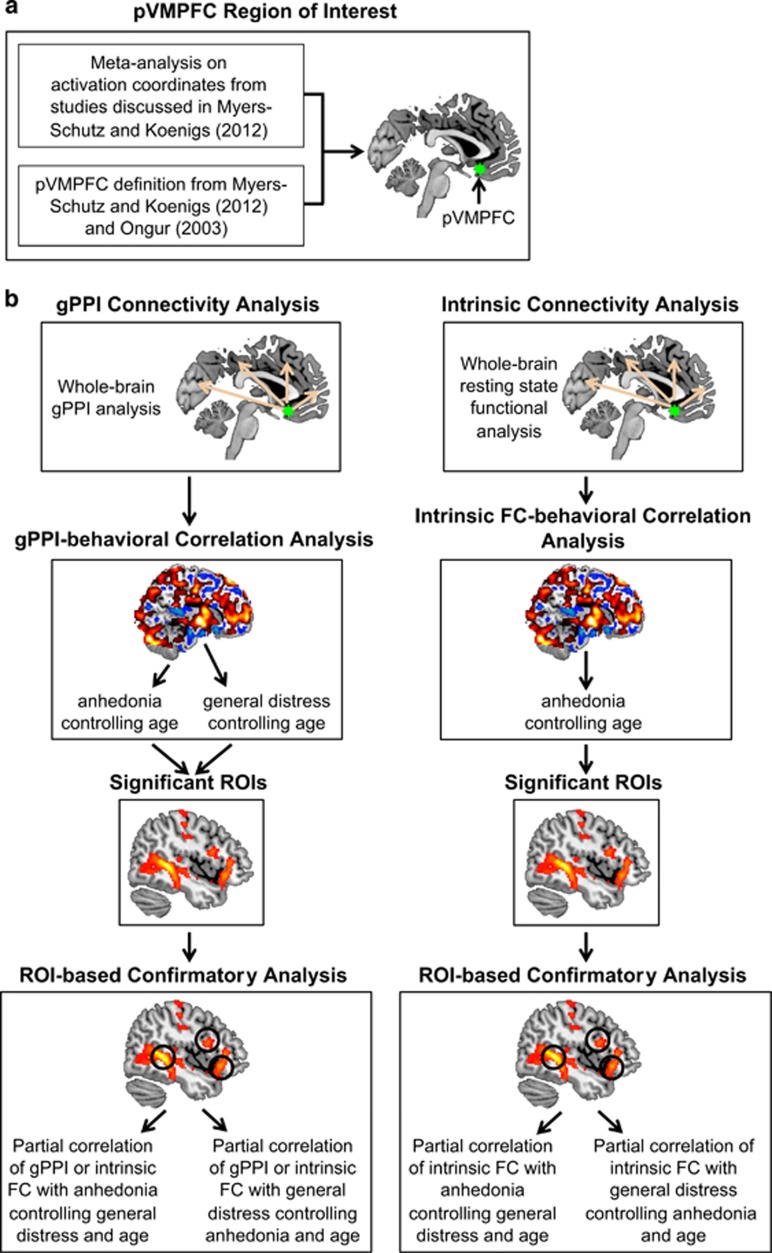
Flow chart depicting the analyses performed in this study. (**a**) A meta-analysis was first conducted to identify a pVMPFC region implicated in mood and anxiety disorders. (**b**) Task-based and intrinsic connectivity related to anhedonia and general distress were then examined in both depressed patients and healthy controls. Confirmatory correlation and partial correlation analyses were used to confirm the robustness of the findings. FC, functional connectivity; gPPI, generalized form of psychophysiological interaction; ROI, region of interest.

The acquisition and preprocessing details of both task and resting-state fMRI data are described in Supplementary Materials. Figure 1 summarizes the analyses conducted in this study.

### Activation analysis

We first confirmed that reward-related regions showed expected task-related brain activation during music listening. Details are available in Supplementary Materials (Supplementary Table S3).

### Connectivity analyses

#### Seed ROIs

Seed ROIs for all connectivity analyses were obtained from a meta-analysis^65, 66, 67^ performed on the studies described in the review article by Myers-Schulz and Koenigs.^32^ Six-millimeter spherical ROIs were created around the peak foci obtained from the GingerALE meta-analysis that was within the pVMPFC (Montreal Neurological Institute (MNI) coordinates: −2, 24, −14) and the perigenual VMPFC (MNI coordinates: −6, 36, −12) regions outlined in Myers-Schulz^32^ and Ongur.^55^ Perigenual VMPFC control analyses highlighted the specificity of results to the pVMPFC (Supplementary Tables S6–S9). Additional details are available in Supplementary Materials.

#### Psychophysiological interaction analyses

A generalized form of psychophysiological interaction^68^ was used to examine task-modulated pVMPFC connectivity with the rest of the brain during Music versus Scrambled conditions. Additional information about generalized form of psychophysiological interaction and individual participant level analysis is available in Supplementary Materials.

For group-level analyses, individual psychophysiological interaction contrast images were entered into three separate two-sample *t*-tests with age as a covariate of no interest: (1) pVMPFC seed only to examine connectivity main effects, (2) pVMPFC seed with a MASQ-AD-PA covariate and (3) pVMPFC seed with a MASQ-GDD covariate. Significant activation clusters were assessed using a voxel-wise statistical height threshold of *P*<0.01, with family-wise error correction at the cluster level *P*<0.01 (*k*=128 voxels) as determined by Monte Carlo simulations.^69^

#### Resting-state connectivity analyses

Regional time series within the seed ROI were extracted from bandpass filtered resting-state fMRI data (0.008–0.10 Hz). Each time series was then submitted into an individual-level fixed-effects analysis under the general linear model framework. A global signal regressor and six motion parameters for each participant were included as covariates of no interest in the model to account for physiological noise and movement-related artifacts.

The relation between anhedonia and pVMPFC resting-state connectivity at the whole-brain level with age as a covariate of no interest was examined using a two-sample *t*-test. Significant activation clusters were assessed using a voxel-wise statistical height threshold of *P*<0.01, with family-wise error correction at the cluster level *P*<0.01.

### Confirmatory correlation and partial correlation analyses

To confirm the robustness of the findings, we further examined the regions that showed a significant relationship between task-modulated pVMPFC connectivity and MASQ-AD-PA and MASQ-GDD scores (that is, generalized form of psychophysiological interaction covariate results) in MDD and healthy control groups. In these regions, both parameter estimates from task-modulated and resting-state connectivity analyses were extracted for each participant. MASQ-AD-PA partial correlations controlling for age, MASQ-GDD partial correlations controlling for age, partial correlations of MASQ-AD-PA controlling for age and MASQ-GDD, as well as partial correlations of MASQ-GDD controlling for age and MASQ-AD-PA were assessed for significance at *P*<0.05 with FDR correction for multiple comparisons.

## Results

### Participant information

The MDD patients had significantly more severe anhedonia (*t*(40)=10.09, *P*<0.001) and general distress (*t*(40)=11.727, *P*<0.001) than healthy controls. The MDD patients had a mean±s.d. HDRS score of 26.57±7.12 with all but four participants in the moderately or severely depressed range, a mean±s.d. MASQ-AD-PA score of 61.81±4.77 (range: 49 to 69), and a mean±s.d. MASQ-GDD score of 38.62±9.38 (range 20 to 54). Healthy controls had a mean±s.d. HDRS score of 0.68±1.32, a mean±s.d. MASQ-AD-PA score of 39.27±9.11 (range: 26 to 57) and a mean±s.d. MASQ-GDD score of 13.95±2.06 (range 12 to 20).

All the patients had a primary diagnosis of current MDD. Seven of 21 patients had a current comorbid Axis I disorder (two dysthymia only, two anxiety only, two dysthymia and anxiety, one dysthymia and anorexia). Those with comorbid diagnoses were equivalent to those without comorbidities on HDRS,^59^ MASQ-AD-PA and MASQ-GDD,^60, 61^ all *P*-values >0.30. HDRS, MASQ-AD-PA and MASQ-GDD did not differ between comorbidity types (that is, no comorbidities, dysthymia-only comorbidity, anxiety-only comorbidity, dysthymia and anxiety comorbidities, dysthymia and anorexia comorbidities), all *P*-values >0.56.

Eight patients were not currently taking psychiatric medication and 13 were taking medications (9 antidepressants only, 3 antidepressants and anxiolytics, 1 antipsychotics only). Those on medication were equivalent to those off medication in HDRS,^59^ MASQ-AD-PA and MASQ-GDD,^60, 61^ all *P*-values >0.24. Furthermore, one-way analyses of variance showed that HDRS, MASQ-AD-PA and MASQ-GDD did not differ depending in medication type, HDRS: *P*=0.08, MASQ-AD-PA: *P*=0.65, MASQ-GDD: *P*=0.30. Additional analyses controlling for comorbidity status and medication use are in Supplementary Materials (Supplementary Figures S4 and S5).

### Music stimulus ratings

A repeated-measures analysis of variance with group as a between-participant factor and the 10 ratings for Music and Scrambled pieces as within-subject factors showed that MDD and healthy controls did not significantly differ in ratings for Music and Scrambled pieces, *F*(1,35)=1.070, *P*>0.30. Follow-up paired *t*-tests on the significant main effects of rating and stimulus type showed that participants found the Music stimuli to be less annoying and more pleasant, calm, relaxed, consonant, peaceful, happy, moving, interesting and familiar in comparison with Scrambled music pieces (Supplementary Table S2). One-sample *t*-tests against 0 (that is, the neutral point) confirmed that participants found the Music stimuli to be pleasant, not annoying, exciting, consonant, peaceful, happy, moving and interesting, all *P*-values <0.001.

### Music pleasantness and anhedonia

MASQ-AD-PA was negatively correlated with Music pleasantness ratings in healthy controls, *r*=−0.515, *P*=0.017, but not in MDD patients, *P*>0.44. No such relations were detected for MASQ-GDD in either group, all *P*-values >0.75.

### Brain activation during music listening

Similar to our previous study,^8^ pleasant Music compared with Scrambled stimuli evoked significant activation in reward- and emotion-related regions including left orbitofrontal cortex BA47, left amygdala/parahippocampal gyrus and bilateral VMPFC (Supplementary Table S3). On the basis of *a priori* hypotheses of NAc activation during music listening,^8, 48, 54^ we conducted an ROI analysis with 6-mm spherical regions centered on the left (MNI coordinates: −9, 9, −8) and right NAc (MNI coordinates: 9, 9, −8), defined using previous meta-analyses.^70, 71^ We found significant activation of the right (*t*(42)=2.396, *P*=0.02), but not left NAc (*P*>0.64). Scrambled stimuli evoked greater activation in bilateral superior temporal gyrus, right inferior frontal gyrus (IFG) and right inferior parietal lobule when compared with Music (Supplementary Table S3).

### Task-modulated pVMPFC functional connectivity during music listening

In MDD, pleasant music evoked significantly greater pVMPFC connectivity with left pallidum extending into caudate and thalamus, right frontal pole and supramarginal gyrus (Table 1). In contrast, healthy controls showed significant pVMPFC connectivity with bilateral superior temporal gyrus and right lateral occipital cortex. Comparisons between groups revealed that controls did not show greater connectivity in comparison with MDD patients, but MDD patients showed greater pVMPFC connectivity with right frontal pole in comparison with healthy controls. Thus, MDD patients showed greater pVMPFC connectivity with frontal lobe regions implicated in planning and higher-order cognition.^72, 73, 74^

### Relation between anhedonia and task-modulated pVMPFC functional connectivity during music listening

**Figure 2:**
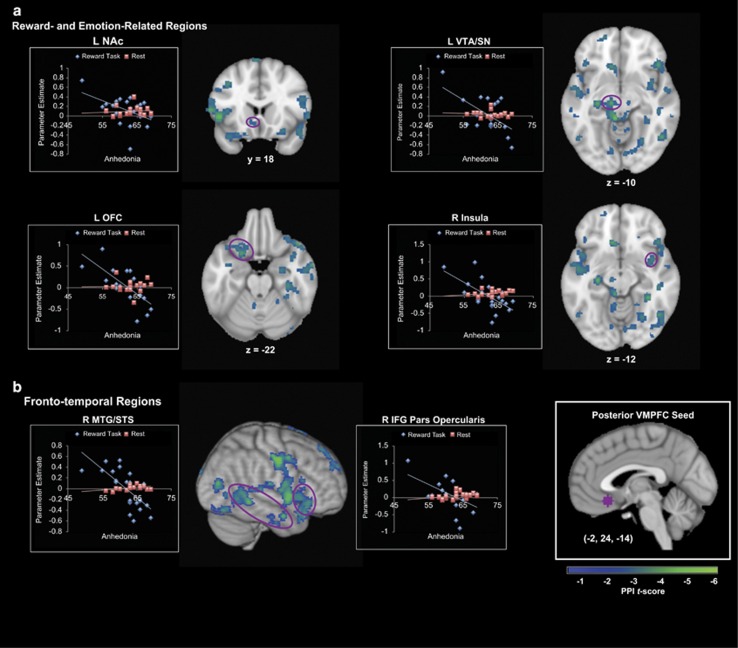
Posterior ventromedial prefrontal cortex (pVMPFC) connectivity in relation to anhedonia in patients with major depressive disorder (MDD). (**a**) Anhedonia was negatively correlated with pVMPFC connectivity during pleasant music listening, but not at rest, in reward- and emotion-related regions including left nucleus accumbens (NAc), left ventral tegmental area/substantia nigra (VTA/SN), left orbitofrontal cortex (OFC) and right mid-insula. (**b**) Anhedonia was also negatively correlated with pVMPFC connectivity during pleasant music listening, but not at rest, with fronto-temporal areas involved in music and speech processing including right middle temporal gyrus/superior temporal sulcus (MTG/STS) and right inferior frontal gyrus (IFG) pars opercularis.

We examined the relation between anhedonia and task-modulated pVMPFC functional connectivity during music listening within and across groups. A significant interaction between MASQ-AD-PA, group and pVMPFC connectivity was driven by negative correlations between pVMPFC connectivity and MASQ-AD-PA in the MDD group and weak positive correlations in the control group (Table 2). Crucially, reward- and emotion-processing, including left NAc, left ventral tegmental area/substantia nigra, left orbitofrontal cortex and right insula showed significant relation differences to anhedonia between the two groups (Figure 2a). Significant interactions were also found in fronto-temporal cortical areas involved in music structure processing, including right IFG pars opercularis and right middle temporal gyrus and superior temporal gyrus (Figure 2b). The pVMPFC connectivity with these reward-related and fronto-temporal cortical regions were significantly negatively correlated with MASQ-AD-PA in the MDD group, while healthy controls showed no significant effects (Table 2). Thus, anhedonia was uniquely related to reduced pVMPFC connectivity with reward- and emotion-related regions, as well as speech and auditory-processing regions in MDD patients.

### Task-modulated pVMPFC functional connectivity during music listening and specificity of links to anhedonia in MDD

**Figure 3:**
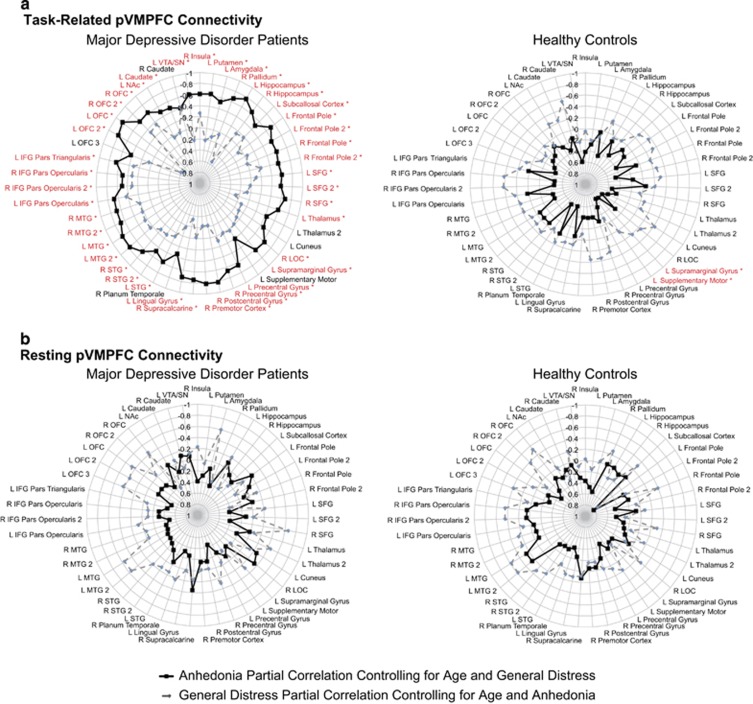
Dissociable effects of anhedonia and general distress on posterior ventromedial prefrontal cortex (pVMPFC) connectivity in major depressive disorder (MDD) patients. (**a**) pVMPFC connectivity during the pleasant music listening task dissociates anhedonia from general distress in patients with MDD. Solid lines depict strength of partial correlations between pVMPFC connectivity and anhedonia after controlling for age and general distress. Dashed lines depict the strength of partial correlations between pVMPFC connectivity and general distress after controlling for age and anhedonia. Links that were significant for anhedonia after controlling for general distress and age after correction for multiple comparisons are shown in red (*P<0.05, FDR corrected). (**b**) The pVMPFC connectivity during resting state did not dissociate anhedonia from general distress in either group.

To further examine whether task-modulated functional pVMPFC connectivity patterns detected above are specific to anhedonia, we performed confirmatory ROI-based analysis using all functional clusters showing significant pVMPFC connectivity related to MASQ-AD-PA that were identified in the whole-brain analysis in either the MDD or control groups. None of these regions showed a significant correlation with MASQ-GDD (*P*>0.05, FDR corrected). We then performed a partial correlation analysis controlling for the effects of age and MASQ-GDD, and found that pVMPFC connectivity with all reward-related and auditory-processing regions except right caudate showed significant relations to anhedonia in MDD patients (*P*<0.05, FDR corrected; Figure 3a).

In healthy controls, only pVMPFC connectivity with left supplementary motor cortex and left supramarginal gyrus was correlated with MASQ-AD-PA after controlling for age and MASQ-GDD (*P*>0.05, FDR corrected); pVMPFC connectivity was not correlated with MASQ-GDD in any of these regions (*P*>0.05, FDR corrected; Figure 3a). Thus, greater anhedonia in MDD patients is associated with weak ability to modulate functional connectivity between pVMPFC and core brain regions involved in reward and emotion processing. Furthermore, this association is specific to anhedonia and not other general symptoms of depression, and is seen in the MDD patients but not in the healthy controls.

### Relation between general distress depressive symptoms and task-modulated pVMPFC functional connectivity during music listening

A significant interaction between general distress depressive symptoms, group and pVMPFC connectivity was observed and driven by both positive and negative correlations between MASQ-GDD and pVMPFC connectivity in MDD, and only negative correlations in healthy controls (Supplementary Figure S2, Supplementary Table S4). In MDD patients, pVMPFC connectivity was positively correlated with MASQ-GDD in the right superior frontal gyrus, and negatively correlated with MASQ-GDD in the right caudate, right subcallosal cortex and right lateral occipital cortex. In the healthy controls, no regions showed a positive correlation between pVMPFC connectivity and MASQ-GDD, but MASQ-GDD was negatively correlated with pVMPFC connectivity with right anterior cingulate cortex and right middle temporal gyrus and superior temporal gyrus. However, pVMPFC connectivity with these regions did not dissociate between MASQ-GDD and MASQ-AD-PA in MDD patients after correcting for multiple comparisons (*P*<0.05, FDR corrected; Supplementary Figure S3A). These results further demonstrate the anatomical specificity of our findings above linking pVMPFC connectivity with the mesolimbic reward system and fronto-temporal auditory-processing regions to anhedonia in the MDD patients (Figure 3a).

### pVMPFC resting-state connectivity not associated with anhedonia in MDD

In the MDD group, MASQ-AD-PA was not positively or negatively correlated with pVMPFC resting-state connectivity in any brain region. In the healthy controls, the MASQ-AD-PA was positively correlated with pVMPFC resting-state connectivity in fronto-temporal cortex (Supplementary Table S6). However, there were no significant differences in the relation between MASQ-AD-PA levels and resting-state pVMPFC connectivity across the groups (Supplementary Table S6). Thus, anhedonia is not associated with resting-state pVMPFC connectivity in MDD.

We also conducted targeted analyses to investigate whether the brain areas that showed a relationship between task-modulated pVMPFC connectivity and MASQ-AD-PA also showed impairments in resting-state functional connectivity. No significant relationship between MASQ-AD-PA and resting-state pVMPFC connectivity was observed in either MDD or healthy control groups (*P*>0.05, FDR corrected; Figure 3b).

Similar analyses were conducted for the regions that showed significant task-modulated relationships between pVMPFC connectivity and MASQ-GDD in MDD and healthy controls. After controlling for age and MASQ-AD-PA, no significant relationship between MASQ-GDD and resting-state pVMPFC connectivity was observed in either group (*P*>0.05, FDR corrected; Supplementary Figure S3B).

### Anatomical specificity in symptom-related functional connectivity

The perigenual subdivision of the VMPFC has also been implicated in depression and anhedonia.^32^ To examine the anatomical specificity of our pVMPFC findings related to anhedonia and general distress, we investigated perigenual VMPFC connectivity in MDD and healthy controls (Supplementary Tables S6–S9). In contrast to the negative correlations observed in MDD patients between task-modulated pVMPFC connectivity and MASQ-AD-PA (Figure 2, Table 2), a positive correlation was found for task-modulated perigenual VMPFC connectivity (Supplementary Table S7). Crucially, the regions that showed a positive correlation between perigenual VMPFC connectivity and MASQ-AD-PA were also significantly correlated with MASQ-GDD, suggesting that perigenual VMPFC connectivity patterns do not distinguish between anhedonia and general distress. Detailed results pertaining to task-modulated and resting-state perigenual VMPFC connectivity in relation to MASQ-AD-PA and MASQ-GDD in MDD and healthy controls are available in Supplementary Materials.

## Discussion

The primary goal of this study was to investigate the differential relationships of anhedonia and general distress symptoms with task-modulated and resting-state pVMPFC functional connectivity in current MDD patients. We also investigated whether these differential relationships can be detected in healthy controls as a secondary aim. Our study demonstrates that in MDD patients, anhedonia, but not general distress, is associated with weak communication between pVMPFC and multiple brain regions important for reward, emotion and auditory processing. In contrast, pVMPFC resting-state connectivity was not associated with anhedonia in current MDD patients. Thus, the dissociation between anhedonia and general distress in pVMPFC connectivity is specific to the context of processing pleasant stimuli and does not appear to be a general feature of intrinsic resting-state brain connectivity in MDD. In addition, these pVMPFC dissociations were specific to current MDD patients and were not seen in the healthy controls. Our findings provide important new insights into the neurobiological basis of anhedonia in affected individuals and highlight its unique relationship with reward- and emotion-related functional circuits.

### Anhedonia in MDD is associated with weak task-modulated pVMPFC connectivity with reward-related regions

Our findings suggest that anhedonia is characterized by a lack of engagement between the pVMPFC and reward-related functional circuits while processing pleasant stimuli. We found that pVMPFC connectivity with core nodes of the mesolimbic reward system is negatively correlated with anhedonia during music listening in current MDD patients. In MDD patients, greater anhedonia was associated with weaker pVMPFC connectivity with the ventral tegmental area/substantia nigra and NAc. The ventral tegmental area/substantia nigra contains dopamine neurons that are central to the reward response,^75^ and has dense reciprocal connections to the NAc.^76^ The NAc is essential for detecting and modulating responses to rewarding stimuli^77^ and has been implicated in both ‘liking' and ‘wanting' components of reward.^78, 79^ Connectivity between the pVMPFC and orbitofrontal cortex, a region important for the hedonic experience of reward, also showed a negative relationship with anhedonia in current MDD patients. The orbitofrontal cortex has dense reciprocal connections with the VMPFC^33, 80^ and is involved in reward value prediction and integration of reward cues.^81^ Crucially, these effects were specific to anhedonia—general distress was not correlated with pVMPFC connectivity with reward-related brain regions, and in fact had minimal impact on pVMPFC connectivity overall. In sum, anhedonia is specifically associated with aberrant functional links that span multiple regions of the mesolimbic reward system and its cortical projections. More generally, our results suggest that distinct abnormalities in functional connectivity underlie anhedonia symptoms in MDD.

### Anhedonia in MDD negatively impacts pVMPFC connectivity with auditory and language regions

In addition to reward-related regions, anhedonia was also negatively correlated with task-modulated pVMPFC connectivity to fronto-temporal areas involved in processing musical structure, including bilateral IFG pars triangularis and pars opercularis as well as bilateral middle temporal gyrus/superior temporal sulcus in those with current MDD. Besides tracking prosodic information,^82^ tonal structure^83^ melodies^84^ and the structural elements of music over time,^85^ these fronto-temporal regions also interact with the mesolimbic reward system.^8, 48, 86^ A study that required neurotypical adults to determine the subjective reward value of individual musical stimuli demonstrated that aesthetic aspects of reward processing result from interactions between the mesolimbic system and secondary auditory cortex.^54^ Furthermore, we previously demonstrated that in neurotypical adults, trait anhedonia is modulated by functional connectivity between core mesolimbic regions and auditory cortex regions.^8^ Taken together, our findings suggest that anhedonia may disrupt the ability to experience pleasure from positive auditory stimuli like music, which requires successful integration of sensory perception with reward-related cognitive and evaluative processes. The relation between anhedonia and the extended reward network observed in our study likely reflects an inability to engage with positively valenced music stimuli, as both groups were able to rate the music stimuli as pleasant. Consistent with this view, anhedonia was negatively correlated with pleasantness ratings in healthy controls, but not in patients. Taken together with our brain imaging findings, these results suggest that while MDD patients are not impaired at rating the pleasantness of musical stimuli, they may experience pleasurable stimuli differently. Further research is needed to examine this question and determine whether the deficits identified here reflect apathy^87^ or reduced interest or motivation to engage with pleasant stimuli.

### Task-specific functional connectivity but not resting-state pVMPFC connectivity is related to anhedonia in MDD

An important question that has not yet been addressed in depression research is whether anhedonia is associated with resting-state brain connectivity or an inability to appropriately modulate brain responses when encountering pleasurable stimuli. Two findings from our study suggest that the effects of anhedonia are task or context specific. First, anhedonia was differentially associated with pVMPFC connectivity when processing pleasant musical stimuli but not during rest. Second, these relations were specific to music stimuli and were not detected when processing scrambled music, the control stimuli used in the task-based fMRI investigation. Thus, anhedonia reflects a lack of engagement between the pVMPFC and reward-related functional circuits when encountering pleasurable stimuli, rather than a constant static deficit in connectivity. Our findings and approach here suggest that longitudinally tracking both task-induced and resting-state changes has the potential to inform how deficits in functional organization might result in aberrant reward processing and anhedonia.

### Limitations and future directions

Although our study represents an important step towards the identification of anhedonia-related functional circuits in MDD, several limitations are important to note. First, the current MDD and the healthy control groups significantly differed in age. However, our analyses were primarily focused on the MDD group and we included age as a covariate of no interest in all the analyses; including this covariate did not affect the task-based connectivity results within either group. Second, although anhedonia was negatively correlated with pleasantness ratings in healthy controls but not in patients, there were no significant differences in music stimulus ratings across groups. Further research is needed to examine how pVMPFC connectivity is differentially related to anhedonia, pleasantness ratings and hedonic value. Third, our sample size was modest so analyses testing the potentially confounding effects of medication status and comorbidity may be underpowered. Finally, including sad stimuli would enable the investigation of differential relationships between pVMPFC-NAc circuits involved in the experience of positively valenced rewarding stimuli versus pVMPFC-amygdala circuits implicated in processing negatively valenced stimuli. In this context, auditory stimuli could have a distinct advantage over the more widely used visual stimuli.^45^ Finally, studies distinguishing different anhedonia subtypes^88, 89^ are important for refining our understanding of how different anhedonia phenotypes are represented in the brain.

## Conclusions

Consistent with the goals of the research domain criteria initiative,^1^ our study provides new insights into the neurobiological basis of anhedonia and highlights its unique relationship with emotion- and reward-processing systems. More specifically, we show that anhedonia in MDD is associated with disrupted communication between the pVMPFC and reward- and emotion-related regions during the processing of positively valenced stimuli. Our results also indicate that in adults with MDD, anhedonia reflects a lack of engagement between the pVMPFC and reward-related functional circuits when contextually appropriate, rather than a constant deficit in connectivity. Thus, probing resting-state connectivity of the pVMPFC may be inadequate for assessing anhedonia-specific effects. Our findings also support the notion that specific psychiatric symptoms in MDD can be linked to distinct neurophysiological pathways.^1^ Finally, our results show that connectivity patterns related to anhedonia and general distress vary between VMPFC subregions, demonstrating the importance of anatomical specificity. In summary, the identification of specific anhedonia-related circuits can facilitate a better understanding of psychopathology and heterogeneity in MDD, as well as more effective tracking of one of the core MDD symptoms.

## Figures and Tables

**Table 1:** Brain regions that showed significant task-modulated functional connectivity to the posterior VMPFC (pVMPFC) during music listening in patients with major depressive disorder (MDD) and healthy controls

	*Size of cluster (voxels)*	*Peak T-score*	*Peak MNI coordinates (mm)*		
			x	y	z
*MDD*					
R frontal pole	280	3.39	24	56	8
L pallidum	478	4.26	−12	0	0
L thalamus		3.58	−4	−4	8
L caudate		3.52	−8	4	4
R supramarginal gyrus	824	3.58	42	−36	42

*Healthy controls*					
R superior temporal gyrus	1500	4.94	62	2	−12
R lateral occipital cortex	149	3.89	42	−82	−8
L superior temporal gyrus	287	3.27	−62	−34	10

*MDD > healthy controls*					
R frontal pole	260	3.25	24	56	10

*Healthy controls > MDD*					
NA					

Abbreviations: L, left; MNI, Montreal Neurological Institute; NA, not applicable; R, right; VMPFC, ventromedial prefrontal cortex.

**Table 2:** Brain regions that showed significant correlations between task-modulated posterior ventromedial prefrontal cortex (pVMPFC) connectivity and anhedonia in patients with major depressive disorder (MDD) and healthy controls

	*Size of cluster (voxels)*	*Peak T-score*	*Peak MNI coordinates (mm)*		
			x	y	z
*MDD*					
Positive					
NA					
Negative					
L middle temporal gyrus	2288	5.73	−62	−40	0
L precentral gyrus	3945	5.25	−64	4	8
L inferior frontal gyrus pars opercularis		4.67	−58	20	8
L putamen		3.76	−30	4	6
L amygdala		3.04	−26	−4	−16
R supracalcarine cortex	1756	5.31	22	−62	16
R premotor cortex	3144	5.19	60	−8	46
R orbitofrontal cortex		4.00	46	26	−8
R hippocampus		3.58	28	−8	−20
R inferior frontal gyrus pars opercularis		3.39	54	20	22
R caudate		2.64	8	8	−2
R insula		3.24	38	4	−12
L hippocampus	1317	4.20	−26	−20	−14
L ventral tegmental area/substantia nigra		4.02	−10	−20	−10
R middle temporal gyrus	2751	4.76	52	−58	−4
R superior temporal gyrus		4.15	50	−32	6
L frontal pole	774	4.49	−22	58	12
L subcallosal cortex	134	2.84	−4	16	−8
L nucleus accumbens		2.60	−12	18	−6
L superior frontal gyrus	160	4.13	−4	14	68
R precentral gyrus	457	3.99	6	−26	52
L lingual gyrus	496	3.52	−18	−56	−4
L orbitofrontal cortex	206	3.95	−20	12	−22
L superior frontal gyrus	208	3.46	−18	−8	66
R frontal pole	179	3.42	20	62	34
L thalamus	383	3.21	−14	−6	6

*Healthy controls*					
Positive					
L cuneus	4227	5.64	−2	−88	32
L supramarginal gyrus	1200	4.67	−64	−50	14
R planum temporale	390	3.73	54	−34	22
L thalamus	238	2.99	−8	−18	−2
L supplementary motor cortex	406	3.43	−10	−6	44
Negative					
NA					

*Interaction*					
Positive MDD, negative healthy controls					
NA					
Negative MDD, positive healthy controls					
R postcentral gyrus	32 467	5.59	60	−8	46
L middle temporal gyrus		5.16	−62	−40	0
R middle temporal gyrus		5.14	52	−58	−4
R pallidum		4.75	20	0	4
L ventral tegmental area/substantia nigra		4.69	−10	−20	−10
L inferior frontal gyrus pars triangularis		4.08	−58	22	8
R inferior frontal gyrus pars opercularis		3.98	54	20	22
R insula		3.72	38	4	−12
R orbitofrontal cortex		3.71	46	24	−10
R superior temporal gyrus		3.54	50	−6	−16
L orbitofrontal cortex		3.29	−34	28	−4
L caudate		3.00	−10	6	8
L superior temporal gyrus		2.93	−50	−10	−12
R superior frontal gyrus	614	4.64	22	−4	72
L frontal pole	617	4.24	−12	66	−2
R lateral occipital cortex	138	4.21	44	−80	18
L orbitofrontal cortex	277	4.12	−22	12	−24
R frontal pole	240	3.82	24	58	20

Abbreviations: L, left; MNI, Montreal Neurological Institute; NA, not applicable; R, right.
