# Supplementary material for: Anhedonia and general distress show dissociable ventromedial prefrontal cortex connectivity in major depressive disorder
Source: Transl Psychiatry. 2016 May 17;6(5):e810–. doi: 10.1038/tp.2016.80 (PMC5070048; doi:10.1038/tp.2016.80)
Supplement: Supplementary Information [file tp201680x1.docx]

**Supplementary Methods**

***fMRI Music Listening Task***

The fMRI music listening task involved active responses to ensure that participants were engaging and attending to the task. The task was programmed in E-Prime (PSTnet Inc., Pittsburgh, PA). Digitized sound files (22,050 sampling rate, 16 bit mono) were presented binaurally using a custom-built magnet compatible system that attenuated scanner noise by approximately 35 dB and the volume was individually set at a comfortable listening level prior to the beginning the study. Loudness level at the participant’s head was approximately 98 dB, and background noise was approximately 63 dB at the subjects’ ears after the attenuation provided by ear inserts and headphones.

Scrambled stimuli were created by randomly drawing 250-350 ms variable-sized excerpts from each music piece, and concatenating and smoothing them with a 30 ms linear cross-fade between excerpts of each piece[^1^](#_ENREF_1). The Scrambled pieces lacked temporal structure and temporally driven expectations[^1^](#_ENREF_1), and the elements that manifest themselves across time are disrupted, including melodic contour, the navigation through tonal key spaces[^2^](#_ENREF_2), and any rhythmic groupings lasting longer than 350 ms. Music and Scrambled pieces retained the same distribution of pitch and loudness as well as the same spectral information over the course of the full excerpt. Fast Fourier Transforms between the Music and Scrambled versions correlated significantly for all selections, *r* = .99, *p* < .001. In a previous study, an independent group of participants rated these classical Music clips as pleasant and their Scrambled counterparts as relatively unpleasant[^3^](#_ENREF_3).

***fMRI Data Acquisition***

Functional images were acquired on a 3T GE Signa scanner using a standard GE whole-head coil (software Lx 8.3). 29 axial slices (4.0 mm thick, 0.9 mm skip) parallel to the AC-PC lines covering the whole brain were imaged using a T2* weighted gradient echo spiral in-out[^4^](#_ENREF_4) pulse sequence (TR = 2.0 s, TE = 30 ms, flip angle = 80^o^, and 1 interleave). The field of view was 200 mm and the matrix size was 64 x 64, providing an in-plane spatial resolution of 3.125 mm. For 2 patients, the resting-state scan had a field of view of 220 mm and an in-plane spatial resolution of 3.4375 mm. To reduce blurring and signal loss arising from field inhomogeneities, an automated high-order shimming method based on spiral acquisitions was used before acquiring functional MRI scans[^5^](#_ENREF_5). High-resolution T1-weighted structural brain images were acquired in the same session to aid in localization of brain activation.

***fMRI Data Analysis***

*Preprocessing*  Data were analyzed using SPM8 software (<http://www.fil.ion.ucl.ac.uk/spm>). The first 2 and 5 volumes for task and resting-state data, respectively, were not analyzed to allow for signal equilibration. Images were realigned to correct for motion, corrected for errors in slice-timing, spatially transformed to standard stereotaxic MNI space, resampled every 2 mm using sinc interpolation and smoothed with a 6 mm full-width half-maximum Gaussian kernel to decrease spatial noise prior to statistical analysis. Translational movement in millimeters (x, y, z) and rotational motion in degrees (pitch, roll, yaw) were calculated based on SPM8 parameters for motion correction of the functional images in each subject. Movement was less than 3 mm translation and 1 degree of rotation in the final sample of 21 depressed patients and 22 healthy controls during the music listening task, as well as for the final sample of 17 depressed patients and 16 healthy controls for the resting-state scan.

*Task-related Activation Analyses* Individual subject analyses were first performed by modeling task-related conditions as well as 6 movement parameters from the preprocessing procedure. Brain activity related to the task conditions (Music, Scrambled) was modeled using boxcar functions convolved with a canonical hemodynamic response function and a temporal dispersion derivative to account for voxel-wise latency differences in hemodynamic response. Low-frequency drifts at each voxel were removed using a high-pass filter (0.5 cycles/min) and serial correlations were accounted for by modeling the fMRI time series as a first-degree autoregressive process. Voxel-wise *t*-statistics maps for each condition were generated for each participant using a general linear model, along with the respective contrast images. Group-level activation was determined using individual subject contrast images and a second-level one-sample *t*-test. In a single group consisting of MDD patients and healthy controls, brain areas that showed greater activation to Music contrasted with Scrambled pieces, as well as Scrambled in contrast to Music pieces, were first identified with grand-mean centered age as a covariate of no interest. All analyses were voxel-wise height thresholded at *p* < .01, with FWE correction at the cluster level *p* < .01 (*k* = 128 voxels) determined by Monte Carlo simulations[^6^](#_ENREF_6).

*Connectivity Analyses*

*pVMPFC Seed Region of Interest* The review by Myers-Schulz and Koenigs^[7](#_ENREF_7)^ contained a total of 40 studies that implicated the pVMPFC and perigenual VMPFC in mood and anxiety. Coordinates were obtained from original studies when possible (34 of 40 studies). MNI coordinates were converted to Talairach space when appropriate using icbm2tal transform[^8^](#_ENREF_8) as implemented by GingerALE 2.3. Using GingerALE 2.3[^9-11^](#_ENREF_9), an ALE meta-analysis was performed on all coordinates obtained from studies in the review and thresholded at *p* < .01 with false-discovery rate (FDR) correction making no assumptions about how the data are correlated[^12^](#_ENREF_12), as well as a minimum volume threshold of 100 mm^3^. ALE meta-analysis results were converted back to MNI space using icbm2tal transform[^8^](#_ENREF_8). Because previous neuroimaging studies[^13-17^](#_ENREF_13), as well as findings from psychopharmacology[^14^](#_ENREF_14)^,^ [^18^](#_ENREF_18), psychotherapy[^19^](#_ENREF_19) and deep-brain stimulation treatment research[^20^](#_ENREF_20)^,^ [^21^](#_ENREF_21) have consistently implicating the pVMPFC in MDD, we focused our analysis in the main text on functional circuits associated with the pVMPFC. Notably, an additional region of interest in the perigenual VMPFC (MNI coordinates: -6, 36, -12) was identified as a control seed region to examine the selectivity of results to the pVMPFC.

*Psychophysiological Interaction Analyses* The “Generalized Form of Context-Dependent Psychophysiological Interactions” SPM toolbox[^22^](#_ENREF_22) was used to perform gPPI. Psychophysiological interaction measures the temporal relation between a given seed region and all other brain voxels after accounting for the common driving influence of task activity on both the seed and target voxel[^23^](#_ENREF_23) as well as the main effect from the signal fluctuations of the seed region. gPPI has the additional flexibility of estimating task-dependent functional connectivity within each task condition for experiments with multiple conditions. Simulation and empirical studies have shown that gPPI is more powerful than the standard PPI implementation in SPM, and is especially well-suited for assessing functional connectivity in block design experiments[^24^](#_ENREF_24).

The individual participant level for gPPI included (1) two regressors for the psychological variables (i.e., the two task conditions of Music and Scrambled), (2) one regressor for the physiological variable (i.e., the time course in the seed region), and (3) two regressors for the psychophysiological interaction term (i.e., a cross-product of each psychological variable with the HRF-deconvolved seed region time course was first constructed and then was convolved back with HRF). Movement parameters and a constant term were also included in the model. The time series for the pVMPFC seed was obtained by extracting the first eigenvariate of the raw voxel time series in the ROI separately for each individual. Contrast images comparing Music and Scrambled PPI terms were created for the seed ROI for each subject. Identical gPPI analyses using the perigenual VMPFC seed were performed as control analyses.

**Supplementary Results**

***Age Confound***

As noted in the Materials and Methods as well as Limitations and Future Directions sections of the main text, the MDD and healthy control groups used for this study significantly differed in age. **Figure S1** depicts the distribution of ages for the MDD and healthy control groups. Because of this significant difference, grand-mean centered age was included as a covariate of no interest in all analyses.

***Controlling for Comorbidity Status and Medication Use***

To examine the effect of comorbidity status and medication use, we coded patients based on their comorbidity type (no comorbidities, *n* = 14; mood only comorbidities, *n* = 2; anxiety only comorbidities, *n* = 2, mood and anxiety comorbidities, *n* = 2, mood and anorexia comorbidities, *n* = 1) as well as their medication use (no medication, *n* = 8; antidepressants only, *n* = 9, antidepressant and anxiolytics, *n* = 3; antipsychotics only, *n* = 1). Similar to analyses examining symptom severity and pVMPFC relationships in MDD presented in the main text (**Figure 2, Figure S2, Table 2, Table S4**), we conducted three separate two-sample *t*-tests with age, comorbidity status and medication use as covariates of no interest: (1) pVMPFC seed only to examine connectivity main effects, (2) pVMPFC seed with anhedonia covariate (MASQ-AD-PA), and (3) pVMPFC seed with a general distress covariate (MASQ-GDD). Task-related pVMPFC connectivity in relation to anhedonia (**Figure S4**) and general distress (**Figure S5**) results were nearly identical to the original analyses highlighted in the main text.

***Task-Modulated Functional Connectivity of the Control Perigenual VMPFC Region***

*Overall Connectivity Profile* In MDD patients, the perigenual VMPFC showed significant connectivity with right MTG and occipital fusiform cortex only (**Table S6**). In healthy controls, the perigenual VMPFC showed significant connectivity with the right planum temporale and left LOC. Comparisons between groups revealed that in comparison to healthy controls, MDD patients showed significantly greater connectivity with the right hippocampus, left temporal occipital fusiform cortex and right intracalcarine cortex, whereas healthy controls did not show greater connectivity than MDD patients.

*Relation to Anhedonia* At the whole-brain level, MDD patients showed a positive correlation between MASQ-AD-PA and perigenual VMPFC connectivity with regions including right superior frontal gyrus and right LOC (**Table S7**). Healthy controls showed a positive correlation between MASQ-AD-PA and perigenual VMPFC connectivity with right middle frontal gyrus and right angular gyrus, as well as a negative correlation between MASQ-AD-PA and perigenual VMPFC connectivity with left frontal pole and left parahippocampal gyrus. There were no significant interactions.

*Relation to General Distress Depressive Symptoms* At the whole-brain level, interactions driven by positive and negative correlations between MASQ-GDD and pVMPFC connectivity in MDD and healthy control groups, respectively, were found in regions including bilateral insula (**Table S8**). MDD patients showed a positive correlation between MASQ-GDD and perigenual VMPFC connectivity with regions including left parahippocampal gyrus, right VMPFC, and right IFG pars triangularis. Healthy controls showed a negative correlation between MASQ-GDD and perigenual VMPFC connectivity with regions including bilateral insula.

***Intrinsic Functional Connectivity of the Control Perigenual VMPFC Region***

At the whole-brain level, intrinsic perigenual VMPFC connectivity was positively correlated with MASQ-AD-PA in right superior frontal gyrus, right precentral gyrus, right cuneal cortex and left planum temporale in the MDD group (**Table S9**). In the healthy control group, perigenual VMPFC connectivity was positively correlated with connectivity in bilateral frontal pole, left parietal operculum cortex, right precentral gyrus, left postcentral gyrus, bilateral LOC and bilateral occipital cortex. No significant interactions were found.

**References**

1. Levitin DJ, Menon V. Musical structure is processed in "language" areas of the brain: a possible role for Brodmann Area 47 in temporal coherence. *NeuroImage* 2003; **20**(4)**:** 2142-2152.

2. Janata P, Birk JL, Van Horn JD, Leman M, Tillmann B, Bharucha JJ. The cortical topography of tonal structures underlying Western music. *Science* 2002; **298**(5601)**:** 2167-2170.

3. Menon V, Levitin DJ. The rewards of music listening: response and physiological connectivity of the mesolimbic system. *NeuroImage* 2005; **28**(1)**:** 175-184.

4. Glover GH, Lai S. Self-navigated spiral fMRI: interleaved versus single-shot. *Magnetic resonance in medicine* 1998; **39**(3)**:** 361-368.

5. Kim DH, Adalsteinsson E, Glover GH, Spielman DM. Regularized higher-order in vivo shimming. *Magnetic resonance in medicine* 2002; **48**(4)**:** 715-722.

6. Nichols T, Hayasaka S. Controlling the familywise error rate in functional neuroimaging: a comparative review. *Statistical methods in medical research* 2003; **12**(5)**:** 419-446.

7. Myers-Schulz B, Koenigs M. Functional anatomy of ventromedial prefrontal cortex: implications for mood and anxiety disorders. *Molecular psychiatry* 2012; **17**(2)**:** 132-141.

8. Lancaster JL, Tordesillas-Gutiérrez D, Martinez M, Salinas F, Evans A, Zilles K*, et al*. Bias between MNI and Talairach coordinates analyzed using the ICBM-152 brain template. *Human brain mapping* 2007; **28**(11)**:** 1194-1205.

9. Eickhoff SB, Laird AR, Grefkes C, Wang LE, Zilles K, Fox PT. Coordinate-based activation likelihood estimation meta-analysis of neuroimaging data: A random-effects approach based on empirical estimates of spatial uncertainty. *Human brain mapping* 2009; **30**(9)**:** 2907-2926.

10. Eickhoff SB, Bzdok D, Laird AR, Kurth F, Fox PT. Activation likelihood estimation meta-analysis revisited. *NeuroImage* 2012; **59**(3)**:** 2349-2361.

11. Turkeltaub PE, Eickhoff SB, Laird AR, Fox M, Wiener M, Fox P. Minimizing within-experiment and within-group effects in activation likelihood estimation meta-analyses. *Human brain mapping* 2012; **33**(1)**:** 1-13.

12. Laird AR, Fox PM, Price CJ, Glahn DC, Uecker AM, Lancaster JL*, et al*. ALE meta-analysis: Controlling the false discovery rate and performing statistical contrasts. *Human brain mapping* 2005; **25**(1)**:** 155-164.

13. Greicius MD, Flores BH, Menon V, Glover GH, Solvason HB, Kenna H*, et al*. Resting-state functional connectivity in major depression: abnormally increased contributions from subgenual cingulate cortex and thalamus. *Biological psychiatry* 2007; **62**(5)**:** 429-437.

14. Mayberg HS, Brannan SK, Tekell JL, Silva JA, Mahurin RK, McGinnis S*, et al*. Regional metabolic effects of fluoxetine in major depression: serial changes and relationship to clinical response. *Biological psychiatry* 2000; **48**(8)**:** 830-843.

15. Keedwell PA, Andrew C, Williams SC, Brammer MJ, Phillips ML. A double dissociation of ventromedial prefrontal cortical responses to sad and happy stimuli in depressed and healthy individuals. *Biological psychiatry* 2005; **58**(6)**:** 495-503.

16. Kumari V, Mitterschiffthaler MT, Teasdale JD, Malhi GS, Brown RG, Giampietro V*, et al*. Neural abnormalities during cognitive generation of affect in treatment-resistant depression. *Biological psychiatry* 2003; **54**(8)**:** 777-791.

17. Matthews SC, Strigo IA, Simmons AN, Yang TT, Paulus MP. Decreased functional coupling of the amygdala and supragenual cingulate is related to increased depression in unmedicated individuals with current major depressive disorder. *Journal of affective disorders* 2008; **111**(1)**:** 13-20.

18. Drevets WC, Bogers W, Raichle ME. Functional anatomical correlates of antidepressant drug treatment assessed using PET measures of regional glucose metabolism. *European neuropsychopharmacology* 2002; **12**(6)**:** 527-544.

19. Siegle GJ, Thompson WK, Collier A, Berman SR, Feldmiller J, Thase ME*, et al*. Toward clinically useful neuroimaging in depression treatment: prognostic utility of subgenual cingulate activity for determining depression outcome in cognitive therapy across studies, scanners, and patient characteristics. *Archives of general psychiatry* 2012; **69**(9)**:** 913-924.

20. Mayberg HS, Lozano AM, Voon V, McNeely HE, Seminowicz D, Hamani C*, et al*. Deep brain stimulation for treatment-resistant depression. *Neuron* 2005; **45**(5)**:** 651-660.

21. Lozano AM, Mayberg HS, Giacobbe P, Hamani C, Craddock RC, Kennedy SH. Subcallosal cingulate gyrus deep brain stimulation for treatment-resistant depression. *Biological psychiatry* 2008; **64**(6)**:** 461-467.

22. McLaren DG, Ries ML, Xu G, Johnson SC. A generalized form of context-dependent psychophysiological interactions (gPPI): A comparison to standard approaches. *NeuroImage* 2012; **61**(4)**:** 1277-1286.

23. Friston KJ, Buechel C, Fink GR, Morris J, Rolls E, Dolan RJ. Psychophysiological and modulatory interactions in neuroimaging. *NeuroImage* 1997; **6**(3)**:** 218-229.

24. Cisler JM, Bush K, Steele JS. A comparison of statistical methods for detecting context-modulated functional connectivity in fMRI. *NeuroImage* 2014; **84**(0)**:** 1042-1052.

**Supplementary Figure Legends**

**Figure S1. Age distributions of major depressive disorder (MDD) and healthy control groups.**

**Figure S2.** **Posterior VMPFC (pVMPFC) connectivity in relation to general distress in patients with major depressive disorder (MDD)**. General distress was negatively correlated with pVMPFC connectivity with right caudate and right subcallosal cortex, and positively correlated with pVMPFC connectivity with right superior frontal gyrus during pleasant music listening but not at rest.

**Figure S3. Dissociable effects of anhedonia and general distress on posterior ventromedial prefrontal cortex (pVMPFC) connectivity in healthy controls.** (**A**) pVMPFC connectivity during the pleasant music listening task dissociates general distress from anhedonia in healthy controls, but not patients with major depressive disorder. Solid lines depict strength of partial correlations between pVMPFC connectivity and anhedonia after controlling for age and general distress. Dashed lines depict the strength of partial correlations between pVMPFC connectivity and general distress after controlling for age and anhedonia. Links that were significant for general distress after controlling for age and anhedonia after correction for multiple comparisons are shown in red (* *p* < .05, FDR-corrected). (**B**) pVMPFC connectivity during resting-state did not dissociate anhedonia from general distress in either group.

**Figure S4**. **Posterior VMPFC (pVMPFC) connectivity in relation to anhedonia in patients with MDD, controlling for comorbidity status, medication use and age.** **(A)** Similar to the original analyses presented in the main text (Figure 2), anhedonia was negatively correlated with pVMPFC connectivity with reward- and emotion-related regions including left nucleus accumbens (NAc), left ventral tegmental area/substantia nigra (VTA/SN), left orbitofrontal cortex (OFC), and right insula during pleasant music listening. **(B)** Anhedonia was also negatively correlated with pVMPFC connectivity with fronto-temporal areas involving music and speech processing including right inferior frontal gyrus (IFG) and right middle temporal gyrus/superior temporal sulcus (MTG/STS).

**Figure S5. Posterior VMPFC (pVMPFC) connectivity in relation to general distress in patients with MDD, controlling for comorbidity status, medication use and age.** Similar to the original analyses presented in Figure S2, general distress was negatively correlated with pVMPFC connectivity with right caudate and right subcallosal cortex during pleasant music listening.

**Table S1. Correlations between the four Mood and Anxiety Symptom Questionnaire (MASQ) scales and the anhedonic depression (AD) factors.** Values on the diagonal in brackets denote Cronbach’s alpha, a measure of internal consistency.

| *N* = 43 | General Distress Anxious Symptoms | Anxious Arousal | General Distress Depressive Symptoms | Anhedonic Depression (AD) | AD Positive Affect | AD Depressive/Low Positive Affect |
| --- | --- | --- | --- | --- | --- | --- |
| General Distress Anxious Symptoms | [.900] | *r* = .754,  *p* < .001 | *r* = .751,  *p* < .001 | *r* = .661,  *p* < .001 | *r* = .547,  *p* < .001 | *r* = .755,  *p* < .001 |
| Anxious Arousal |  | [.830] | *r* = .582,  *p* < .001 | *r* = .567,  *p* < .001 | *r* = .456,  *p* = .002 | *r* = .671,  *p* < .001 |
| General Distress Depressive Symptoms |  |  | [.972] | *r* = .881,  *p* < .001 | *r* = .774,  *p* < .001 | *r* = .926,  *p* < .001 |
| Anhedonic Depression (AD) |  |  |  | [.964] | *r* = .967,  *p* < .001 | *r* = .898,  *p* < .001 |
| AD Positive Affect |  |  |  |  | [.962] | *r* = .758,  *p* < .001 |
| AD Depressive/Low Positive Affect |  |  |  |  |  | [.915] |

**Table S2. Mean stimulus ratings.** Ratings were conducted on a 9-point Likert scale (-4 for the first construct listed to +4 for the second construct listed). Reported ratings were pooled across both groups as depressed and control groups did not significantly differ in their ratings. Follow-up paired *t*-tests showed that Music pieces were viewed, in general, as more positive than Scrambled pieces.

|  | Music Rating  + SD | Scrambled Rating + SD | Music vs. Scrambled |
| --- | --- | --- | --- |
| Unpleasant – Pleasant | 1.923 + 0.975 | -1.951 + 1.309 | *t*(36) = 16.421,  *p* < .001 |
| Annoying – Not annoying | 1.860 + 1.187 | -2.180 + 1.234 | *t*(36) = 15.528,  *p* < .001 |
| Excited – Calm | -0.829 + 1.079 | -1.509 + 0.896 | *t*(36) = 3.508,  *p* = .001 |
| Tense – Relaxed | 0.203 + 1.118 | -1.802 + 1.118 | *t*(36) = 9.726,  *p* < .001 |
| Dissonant – Consonant | 1.626 + 1.330 | -2.257 + 1.230 | *t*(36) = 11.918,  *p* < .001 |
| Angry – Peaceful | 0.740 + 0.883 | -0.605 + 0.785 | *t*(36) = 8.753,  *p* < .001 |
| Happy – Sad | -0.870 + 0.709 | 0.018 + 0.790 | *t*(36) = -4.898,  *p* < .001 |
| Moving – Unmoving | -1.671 + 0.979 | 1.311 + 1.545 | *t*(36) = -12.582,  *p* < .001 |
| Boring – Interesting | 1.932 + 0.866 | -0.414 + 1.651 | *t*(36) = 9.360,  *p* < .001 |
| Unfamiliar – Familiar | 1.478 + 0.973 | -1.797 + 1.430 | *t*(36) = 16.271,  *p* < .001 |

**Table S3. Brain areas in depressed patients and healthy controls that showed significant activation during Music, in comparison to Scrambled pieces, as well as Scrambled pieces, in comparison to Music.**

|  | **Size of Cluster (voxels)** | **Peak  *T*-Score** | **Peak MNI Coordinates (mm)** | | |
| --- | --- | --- | --- | --- | --- |
|  |  |  | **X** | **Y** | **Z** |
| **Music - Scrambled** | |  |  |  |  |
| R Paracingulate gyrus | 1978 | 4.34 | 8 | 38 | -6 |
| L Paracingulate gyrus |  | 3.99 | -10 | 50 | 14 |
| R Perigenual VMPFC |  | 3.63 | 2 | 40 | -6 |
| L VMPFC |  | 3.57 | -4 | 54 | -16 |
| L Orbitofrontal cortex |  | 3.05 | -26 | 20 | -22 |
| L Amygdala |  | 2.77 | -28 | 0 | -30 |
| L Superior parietal lobule | 248 | 3.23 | -30 | -46 | 44 |
| L Temporal occipital fusiform cortex | 148 | 3.51 | -42 | -46 | -18 |
|  |  |  |  |  |  |
| **Scrambled - Music** | | |  |  |  |
| R Superior temporal gyrus | 3441 | 6.13 | 68 | -18 | 2 |
| L Superior temporal gyrus | 1776 | 6.04 | -52 | -12 | -2 |
| R Inferior parietal lobule | 3342 | 4.79 | 48 | -50 | 50 |
| R Inferior frontal gyrus pars opercularis | 158 | 3.35 | 46 | 12 | 18 |

**Table S4. Brain regions that showed significant correlations between task-modulated posterior ventromedial prefrontal cortex (pVMPFC) connectivity and general distress in patients with major depressive disorder (MDD) and healthy controls.**

|  | **Size of Cluster (voxels)** | **Peak  *T*-Score** | **Peak MNI Coordinates (mm)** | | |  |
| --- | --- | --- | --- | --- | --- | --- |
|  |  |  | **X** | **Y** | **Z** | |
| **MDD** |  |  |  |  |  | |
| Positive |  |  |  |  |  | |
| R Superior Frontal Gyrus | 130 | 2.84 | 8 | 28 | 56 | |
| Negative |  |  |  |  |  | |
| R Caudate | 757 | 3.52 | 8 | 8 | 2 | |
| R Subcallosal Cortex |  | 3.43 | 2 | 12 | -2 | |
| R Lateral Occipital Cortex | 321 | 3.60 | 46 | -84 | 10 | |
|  |  |  |  |  |  | |
| **Healthy Controls** |  |  |  |  |  | |
| Positive |  |  |  |  |  | |
| n/a |  |  |  |  |  | |
| Negative |  |  |  |  |  | |
| L Lateral Occipital Cortex | 849 | 4.92 | -42 | -84 | 2 | |
| L Postcentral Gyrus | 304 | 3.46 | -54 | -10 | 32 | |
| R Occipital Pole | 1208 | 4.28 | 32 | -96 | 6 | |
| R Occipital Pole | 198 | 4.14 | 4 | -98 | -8 | |
| R Anterior Cingulate Cortex | 204 | 3.82 | 2 | 32 | 26 | |
| R Middle Temporal Gyrus | 152 | 3.62 | 60 | -28 | -4 | |
| R Superior Temporal Gyrus |  | 3.01 | 68 | -12 | 8 | |
|  |  |  |  |  |  | |
| **Interaction** | | |  |  |  | |
| Positive MDD, Negative Healthy Controls | | | | | | |
| L Lateral Occipital Cortex | 739 | 4.92 | -42 | -84 | 2 | |
| L Precentral Gyrus | 189 | 3.10 | -56 | -4 | 40 | |
| R Anterior Cingulate Cortex | 262 | 3.85 | 2 | 32 | 26 | |
| R Occipital Pole | 149 | 3.84 | 4 | -98 | -8 | |
| R Lateral Occipital Cortex | 768 | 3.74 | 48 | -80 | -8 | |
| Negative MDD, Positive Healthy Controls | | | | | | |
| n/a |  |  |  |  |  | |

**Table S5. Brain regions that showed significant correlations between intrinsic resting-state posterior ventromedial prefrontal cortex (pVMPFC) connectivity and anhedonia in patients with major depressive disorder (MDD) and healthy controls.**

|  | **Size of Cluster (voxels)** | **Peak  *T*-Score** | **Peak MNI Coordinates (mm)** | | |  |
| --- | --- | --- | --- | --- | --- | --- |
|  |  |  | **X** | **Y** | **Z** | |
| **MDD** |  |  |  |  |  | |
| Positive |  |  |  |  |  | |
| n/a |  |  |  |  |  | |
| Negative |  |  |  |  |  | |
| n/a |  |  |  |  |  | |
|  |  |  |  |  |  | |
| **Healthy Controls** |  |  |  |  |  | |
| Positive |  |  |  |  |  | |
| R Planum Temporale | 1508 | 6.18 | 58 | -30 | 12 | |
| R Superior Temporal Gyrus |  | 4.66 | 68 | -14 | 6 | |
| R Putamen |  | 4.02 | 30 | -2 | 0 | |
| R Orbitofrontal Cortex |  | 3.98 | 36 | 26 | -6 | |
| L Planum Temporale | 418 | 4.17 | -44 | -10 | -6 | |
| L Inferior Frontal Gyrus Pars Opercularis |  | 3.28 | -50 | 16 | 10 | |
| L Frontal Pole | 1005 | 4.16 | -40 | 46 | 28 | |
| L Lingual Gyrus | 208 | 3.64 | -8 | -56 | -4 | |
| Negative |  |  |  |  |  | |
| n/a |  |  |  |  |  | |
|  |  |  |  |  |  | |
| **Interaction** | | |  |  |  | |
| Positive MDD, Negative Healthy Controls | | | | | | |
| n/a |  |  |  |  |  | |
| Negative MDD, Positive Healthy Controls | | | | | | |
| n/a |  |  |  |  |  | |

**Table S6. Brain regions that showed significant task-modulated functional connectivity with the perigenual VMPFC control region in patients with major depressive disorder (MDD) and healthy controls.**

|  | **Size of Cluster (voxels)** | **Peak  *T*-Score** | **Peak MNI Coordinates (mm)** | | |
| --- | --- | --- | --- | --- | --- |
|  |  |  | **X** | **Y** | **Z** |
| **MDD** |  |  |  |  |  |
| R Middle Temporal Gyrus | 140 | 2.70 | 56 | -48 | -6 |
| R Occipital Fusiform Cortex | 136 | 3.04 | -30 | -66 | -18 |
|  |  |  |  |  |  |
| **Healthy Controls** |  |  |  |  |  |
| R Planum Temporale | 214 | 3.77 | 62 | -22 | 12 |
| L Lateral Occipital Cortex | 147 | 3.34 | -32 | -64 | 42 |
|  |  |  |  |  |  |
| **MDD > Healthy Controls** |  |  |  |  |  |
| R Hippocampus | 164 | 3.85 | 36 | -30 | -12 |
| L Temporal Occipital Fusiform Cortex | 322 | 3.62 | -42 | -60 | -18 |
| R Intracalcarine Cortex | 267 | 3.12 | 22 | -62 | 8 |
|  |  |  |  |  |  |
| **Healthy Controls > MDD** |  |  |  |  |  |
| n/a |  |  |  |  |  |

**Table S7. Brain regions that showed significant correlations between task-modulated perigenual ventromedial prefrontal cortex connectivity and anhedonia in patients with major depressive disorder (MDD) and healthy controls.**

|  | **Size of Cluster (voxels)** | **Peak  *T*-Score** | **Peak MNI Coordinates (mm)** | | |  |
| --- | --- | --- | --- | --- | --- | --- |
|  |  |  | **X** | **Y** | **Z** | |
| **MDD** |  |  |  |  |  | |
| Positive |  |  |  |  |  | |
| R Superior Frontal Gyrus | 205 | 4.64 | 14 | 24 | 66 | |
| R Lateral Occipital Cortex | 149 | 3.03 | 46 | -76 | -16 | |
| Negative |  |  |  |  |  | |
| n/a |  |  |  |  |  | |
|  |  |  |  |  |  | |
| **Healthy Controls** |  |  |  |  |  | |
| Positive |  |  |  |  |  | |
| R Middle Frontal Gyrus | 222 | 3.36 | 58 | 26 | 30 | |
| R Angular Gyrus | 301 | 3.23 | 56 | -54 | 24 | |
| Negative |  |  |  |  |  | |
| L Frontal Pole | 159 | 2.99 | -6 | 58 | 12 | |
| L Parahippocampal Gyrus | 153 | 3.57 | -16 | -30 | -20 | |
|  |  |  |  |  |  | |
| **Interaction** | | |  |  |  | |
| Positive MDD, Negative Healthy Controls | | | | | | |
| n/a |  |  |  |  |  | |
| Negative MDD, Positive Healthy Controls | | | | | | |
| n/a |  |  |  |  |  | |

**Table S8. Brain regions that showed significant correlations between task-modulated perigenual ventromedial prefrontal cortex connectivity and general distress in patients with major depressive disorder (MDD) and healthy controls.**

|  | **Size of Cluster (voxels)** | **Peak  *T*-Score** | **Peak MNI Coordinates (mm)** | | |  |
| --- | --- | --- | --- | --- | --- | --- |
|  |  |  | **X** | **Y** | **Z** | |
| **MDD** |  |  |  |  |  | |
| Positive |  |  |  |  |  | |
| L Parahippocampal Gyrus | 15256 | 5.99 | -18 | -14 | -26 | |
| R Precentral Gyrus | 5776 | 5.11 | 24 | -16 | 72 | |
| R VMPFC | 142 | 3.48 | 4 | 46 | -16 | |
| R Lateral Occipital Cortex | 805 | 4.59 | 54 | -70 | 6 | |
| R Occipital Pole | 137 | 4.45 | 4 | -92 | 36 | |
| R Inferior Frontal Gyrus Pars Triangularis | 206 | 4.41 | 54 | 32 | -2 | |
| R Frontal Pole | 244 | 4.17 | 18 | 62 | 34 | |
| L Postcentral Gyrus | 1788 | 4.15 | -20 | -42 | 64 | |
| L Frontal Pole | 254 | 3.99 | -46 | 44 | 10 | |
| L Posterior Cingulate Cortex | 271 | 3.22 | -2 | -42 | 20 | |
| Negative |  |  |  |  |  | |
| n/a |  |  |  |  |  | |
|  |  |  |  |  |  | |
| **Healthy Controls** |  |  |  |  |  | |
| Positive |  |  |  |  |  | |
| n/a |  |  |  |  |  | |
| Negative |  |  |  |  |  | |
| L Lateral Occipital Cortex | 615 | 5.16 | -44 | -82 | -6 | |
| L Precentral Gyrus | 484 | 3.74 | -58 | 4 | 18 | |
| R Lateral Occipital Cortex | 1914 | 4.18 | 48 | -76 | -2 | |
| L Temporal Pole | 546 | 4.16 | -50 | 16 | -14 | |
| L Insula |  | 3.88 | -40 | -4 | -4 | |
| R Temporal Pole | 292 | 3.81 | 32 | 6 | -48 | |
| R Insula | 311 | 3.51 | 44 | -10 | 6 | |
| R Occipital Pole | 133 | 3.53 | 22 | -96 | -10 | |
|  |  |  |  |  |  | |
| **Interaction** | | |  |  |  | |
| Positive MDD, Negative Healthy Controls | | | | | | |
| L Lateral Occipital Cortex | 6780 | 5.68 | -44 | -82 | -6 | |
| L Precentral Gyrus | 610 | 3.95 | -58 | 4 | 18 | |
| L Insula | 1142 | 4.58 | -40 | -2 | -2 | |
| R Temporal Pole | 500 | 4.35 | 34 | 8 | -48 | |
| R Insula | 831 | 4.18 | 44 | -10 | 6 | |
| L Frontal Pole | 150 | 4.01 | -22 | 66 | -2 | |
| L Precentral Gyrus | 154 | 3.75 | -24 | -22 | 70 | |
| R Precentral Gyrus | 135 | 3.71 | 66 | 4 | 8 | |
| L Superior Parietal Lobule | 178 | 3.64 | -24 | -56 | 56 | |
| R Inferior Parietal Lobule | 132 | 3.60 | 62 | -46 | 14 | |
| R Precentral Gyrus | 226 | 3.51 | 4 | -18 | 52 | |
| Negative MDD, Positive Healthy Controls | | | | | | |
| n/a |  |  |  |  |  | |

**Table S9. Brain regions that showed significant correlations between intrinsic resting-state perigenual ventromedial prefrontal cortex connectivity and anhedonia in patients with major depressive disorder (MDD) and healthy controls.**

|  | **Size of Cluster (voxels)** | **Peak  *T*-Score** | **Peak MNI Coordinates (mm)** | | |  |
| --- | --- | --- | --- | --- | --- | --- |
|  |  |  | **X** | **Y** | **Z** | |
| **MDD** |  |  |  |  |  | |
| Positive |  |  |  |  |  | |
| R Superior Frontal Gyrus | 338 | 4.44 | -2 | 42 | 40 | |
| R Precentral Gyrus | 170 | 4.11 | 54 | -6 | 34 | |
| R Cuneal Cortex | 367 | 4.10 | 6 | -88 | 24 | |
| L Planum Temporale | 232 | 3.45 | -64 | -28 | 14 | |
| Negative |  |  |  |  |  | |
| n/a |  |  |  |  |  | |
|  |  |  |  |  |  | |
| **Healthy Controls** |  |  |  |  |  | |
| Positive |  |  |  |  |  | |
| R Precentral Gyrus | 5698 | 5.10 | 54 | -8 | 42 | |
| L Frontal Pole | 168 | 4.48 | -16 | 46 | 46 | |
| L Lateral Occipital Cortex | 330 | 3.28 | -40 | -72 | -2 | |
| L Parietal Operculum Cortex | 1951 | 4.37 | -52 | -32 | 26 | |
| L Postcentral Gyrus | 200 | 4.34 | -62 | -14 | 38 | |
| R Lateral Occipital Cortex | 680 | 3.86 | 26 | -64 | 48 | |
| R Occipital Cortex | 247 | 3.71 | 8 | -94 | 28 | |
| L Occipital Pole | 216 | 3.68 | -28 | -92 | 0 | |
| R Frontal Pole | 165 | 3.47 | 50 | 50 | 8 | |
| R Lateral Occipital Cortex | 131 | 3.28 | 34 | -84 | 4 | |
| Negative |  |  |  |  |  | |
| n/a |  |  |  |  |  | |
|  |  |  |  |  |  | |
| **Interaction** | | |  |  |  | |
| Positive MDD, Negative Healthy Controls | | | | | | |
| n/a |  |  |  |  |  | |
| Negative MDD, Positive Healthy Controls | | | | | | |
| n/a |  |  |  |  |  | |
