# Supplementary figures and images for: Anhedonia and general distress show dissociable ventromedial prefrontal cortex connectivity in major depressive disorder
Source: Transl Psychiatry. 2016 May 17;6(5):e810–. doi: 10.1038/tp.2016.80 (PMC5070048; doi:10.1038/tp.2016.80)

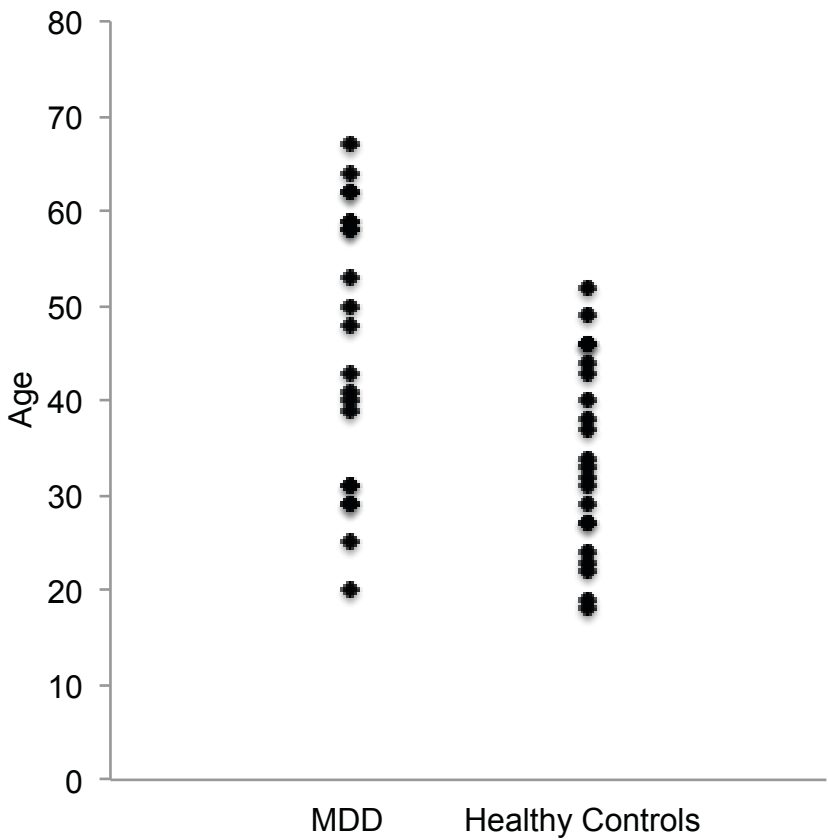

Supplement: Supplementary Figure 1 [file tp201680x2.pdf]

## R Caudate

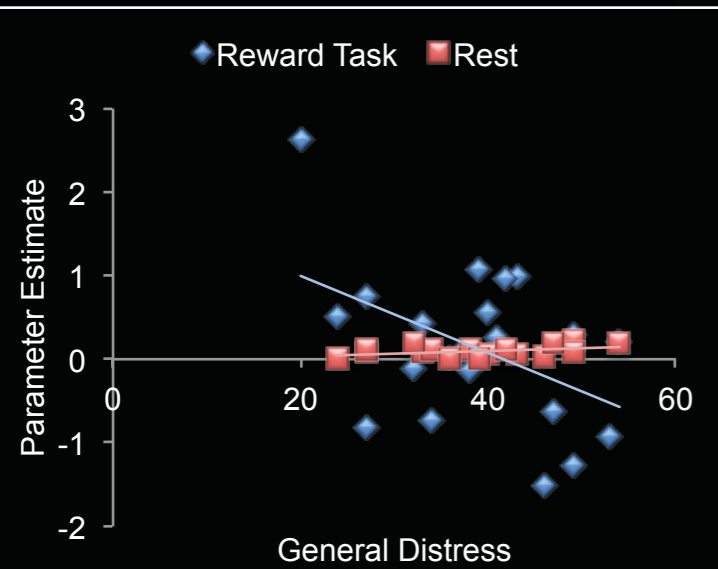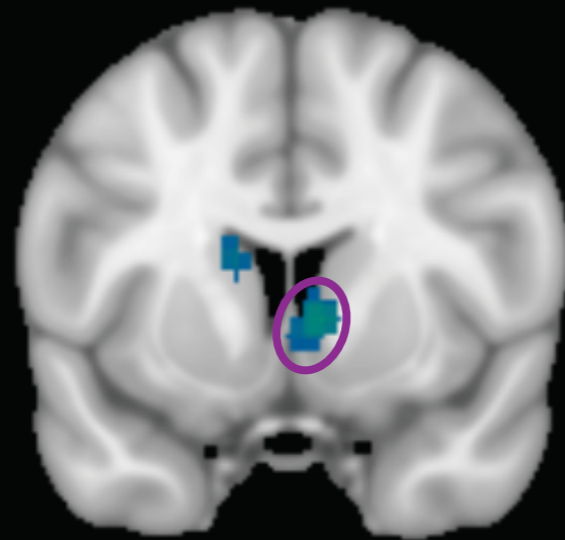

$y = 8$

## R Subcallosal Cortex

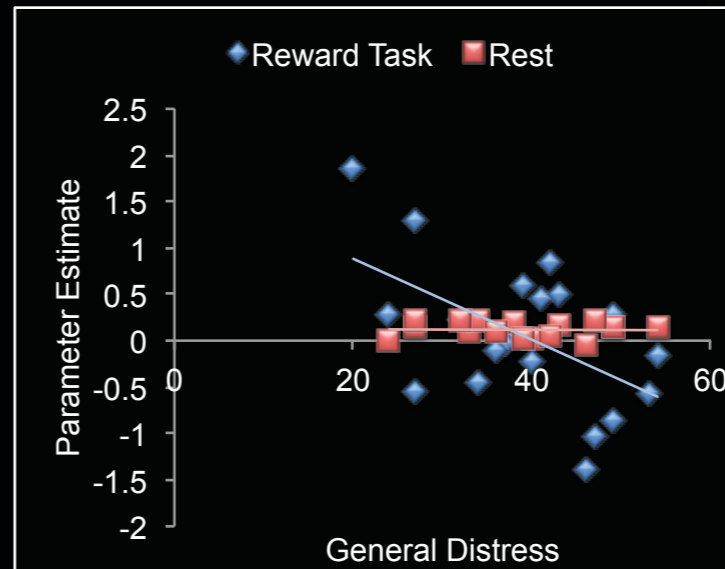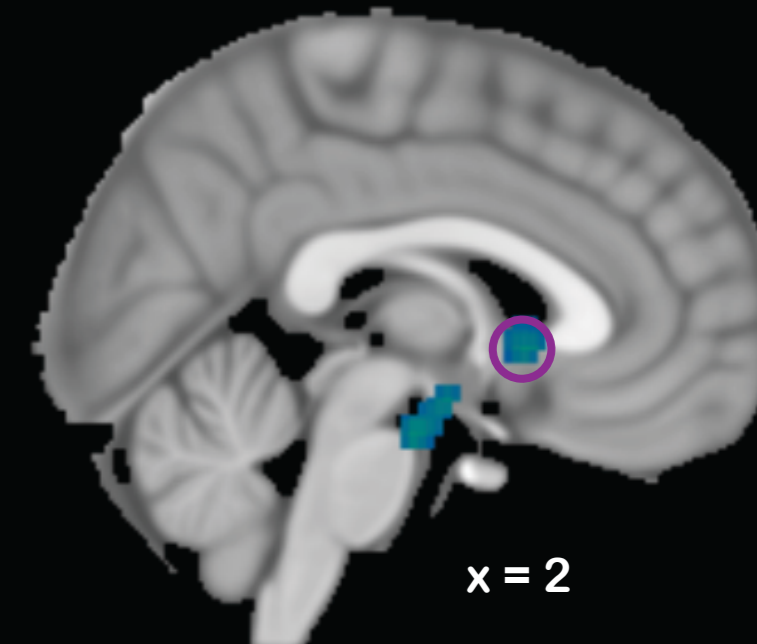

$x = 2$

## R Superior Frontal Gyrus

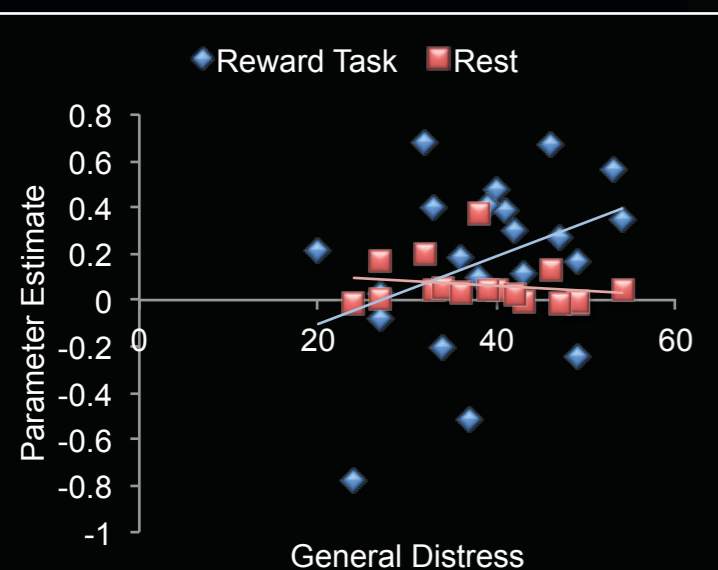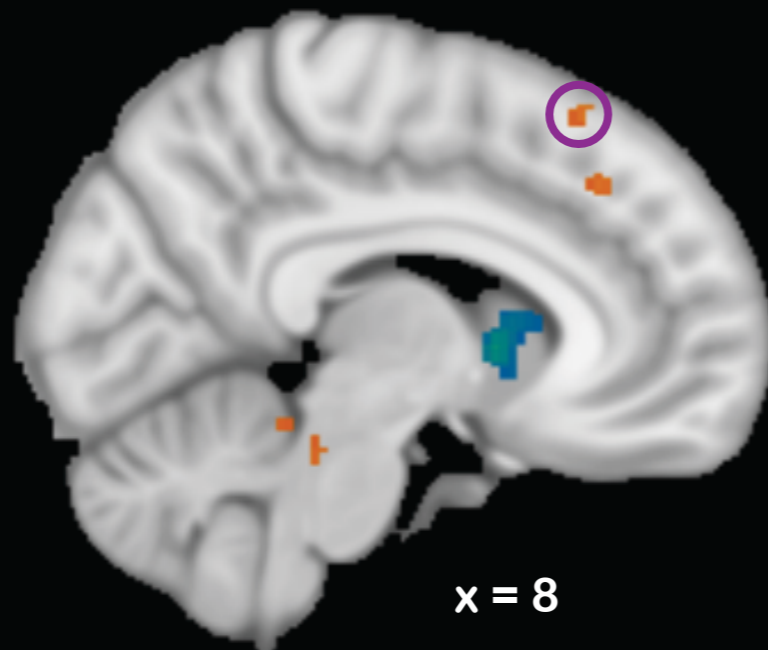

$x = 8$

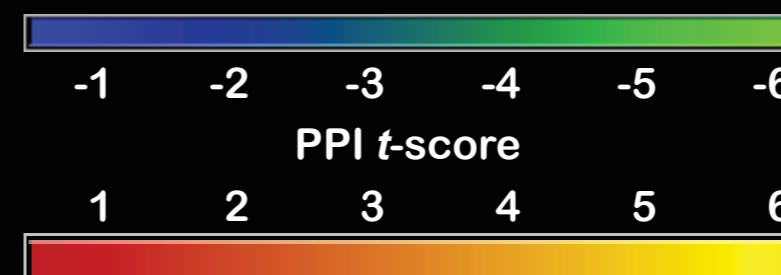

## Posterior VMPFC Seed

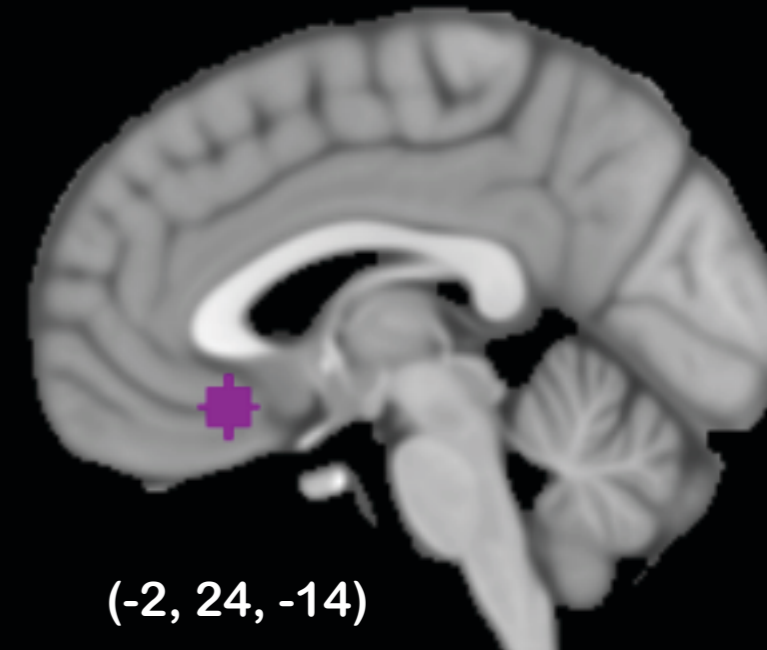

$(-2, 24, -14)$

Supplement: Supplementary Figure 2 [file tp201680x3.pdf]

A. Reward- and Emotion-Related Regions

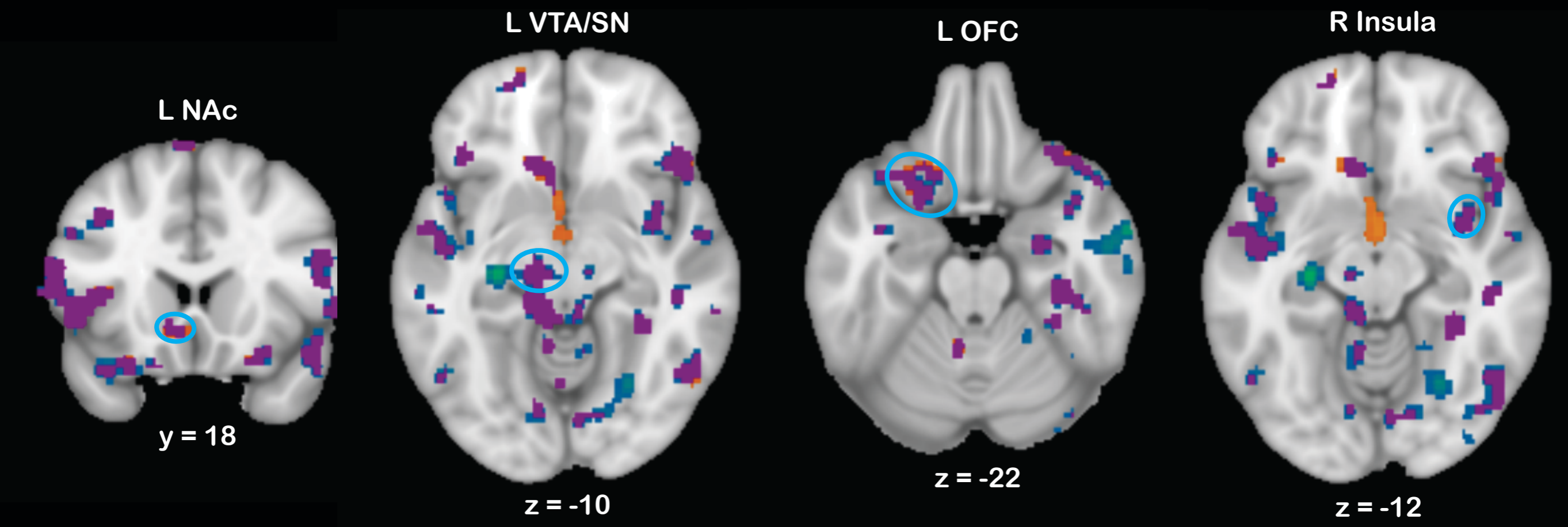

B. Fronto-temporal Regions

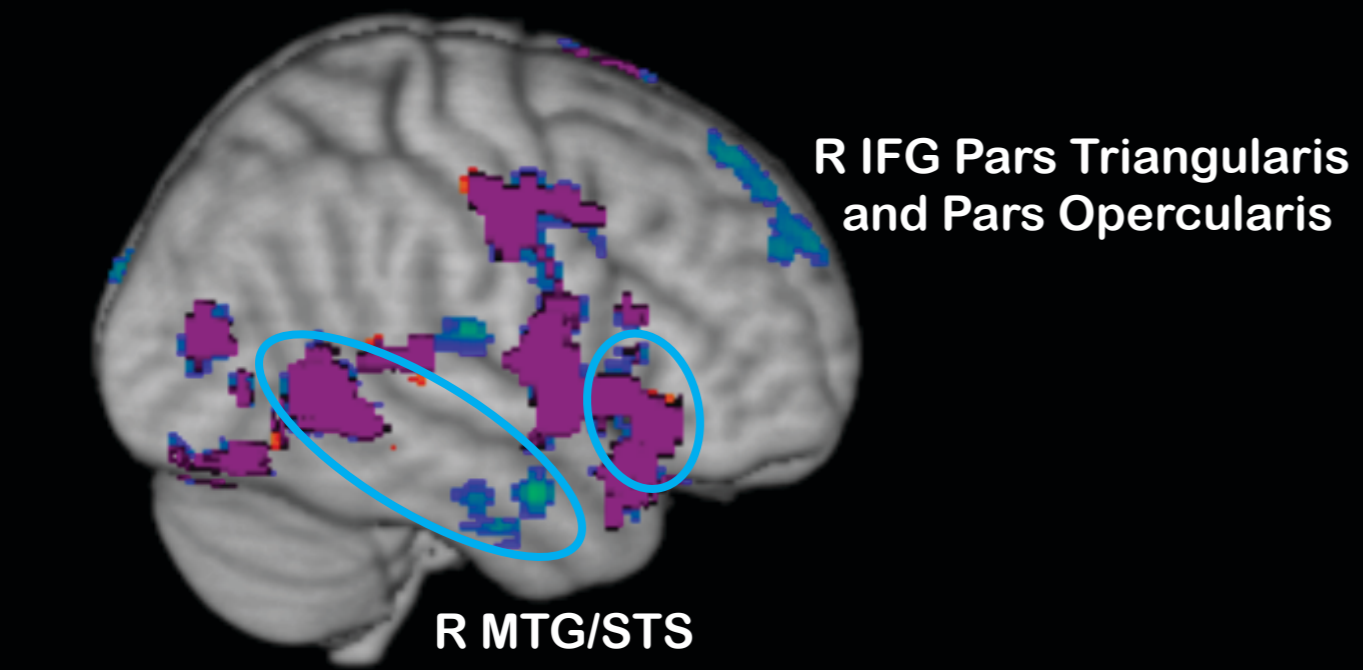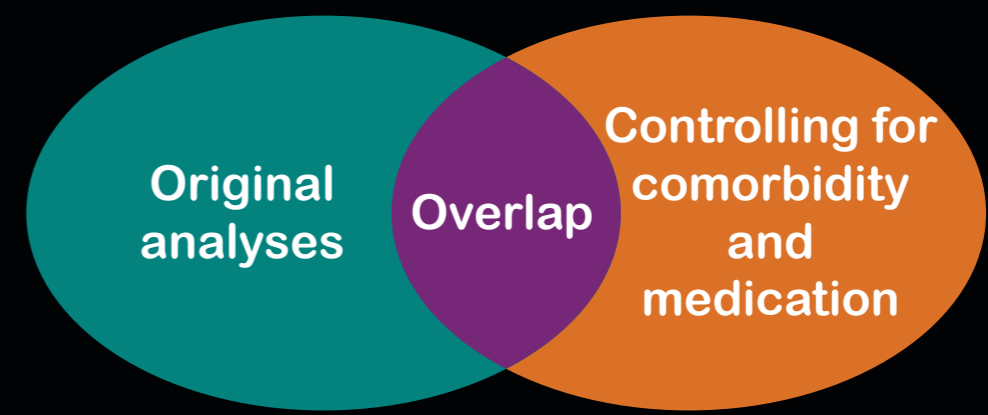

Supplement: Supplementary Figure 4 [file tp201680x5.pdf]

**R Caudate**

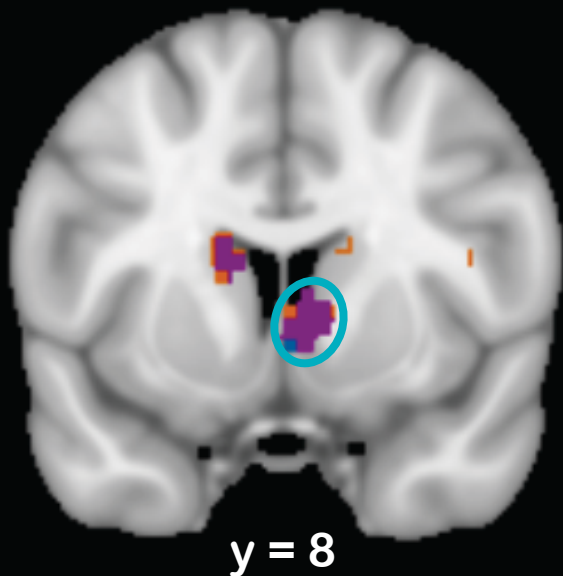

**R Subcallosal Cortex**

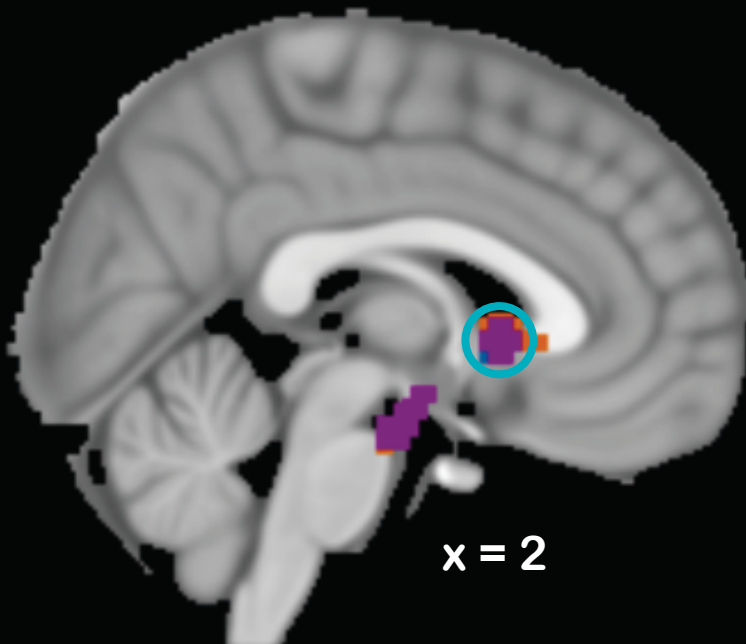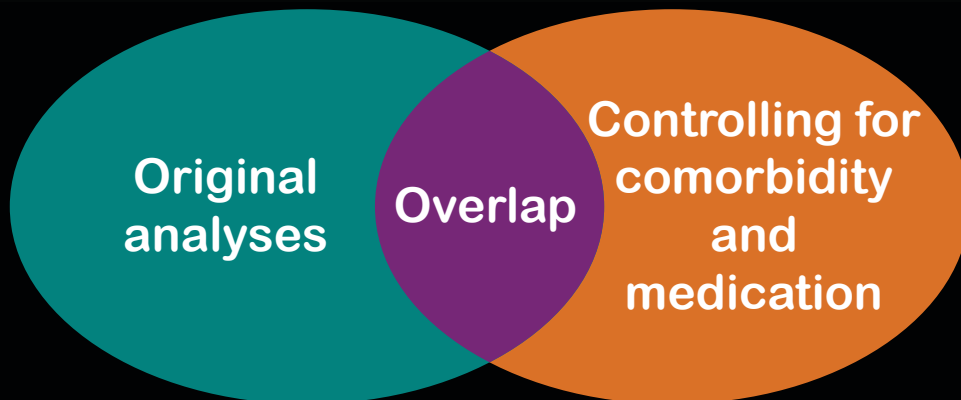

Supplement: Supplementary Figure 5 [file tp201680x6.pdf]
