# Supplementary material for: Anhedonia and general distress show dissociable ventromedial prefrontal cortex connectivity in major depressive disorder
Source: Transl Psychiatry. 2016 May 17;6(5):e810–. doi: 10.1038/tp.2016.80 (PMC5070048; doi:10.1038/tp.2016.80)
Supplement: Supplementary Figure 3 [file tp201680x4.pdf]

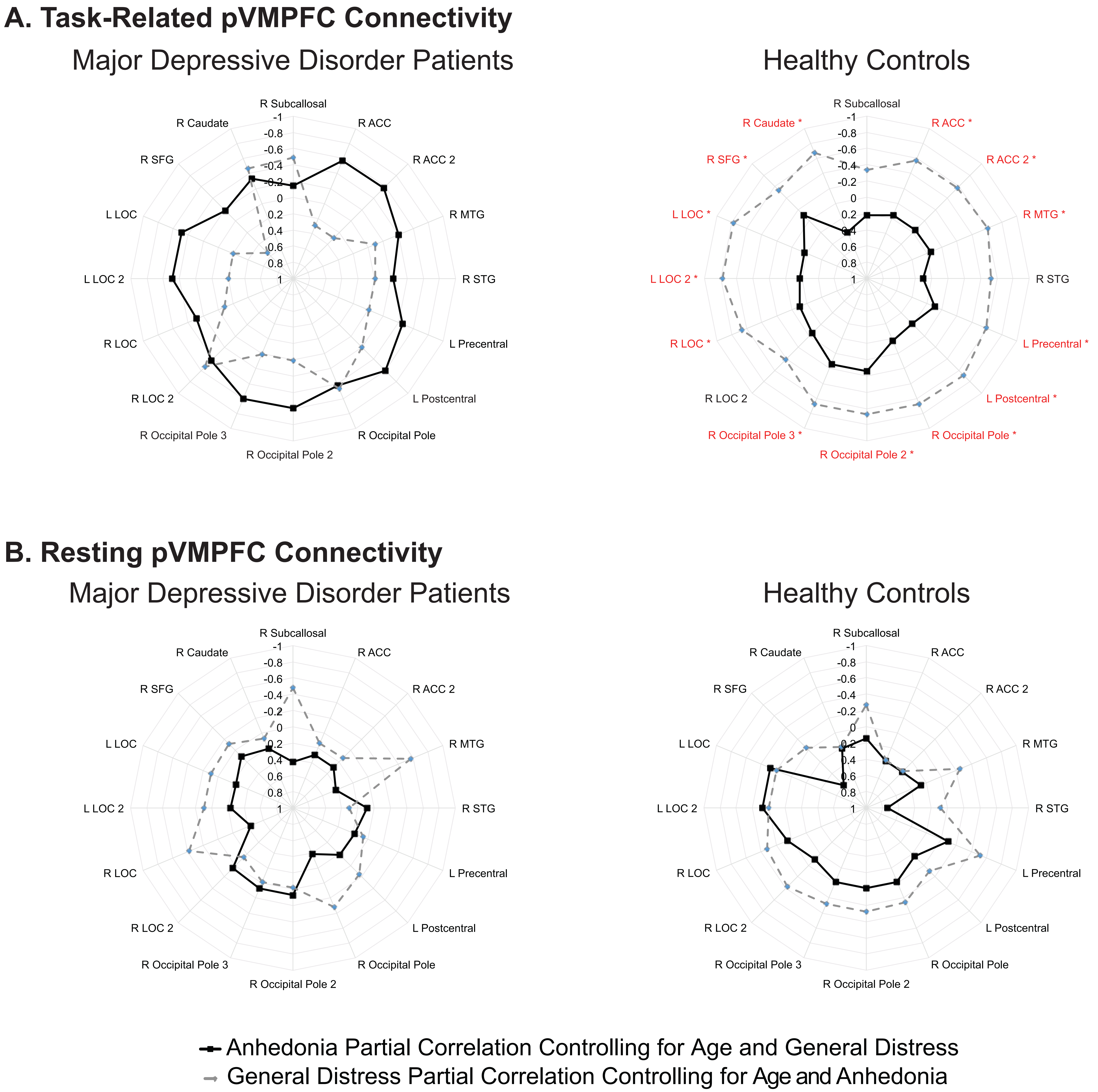
A. Task-Related pVMPFC Connectivity
Major Depressive Disorder Patients
Healthy Controls
R Subcallosal
R ACC
R ACC 2
R MTG
R STG
L Precentral
L Postcentral
R Occipital Pole
R Occipital Pole 2
R Occipital Pole 3
R LOC 2
R LOC
L LOC 2
L LOC
R SFG
R Caudate
R Caudate *
R ACC *
R ACC 2 *
R SFG *
R MTG *
L LOC *
L LOC 2 *
R LOC *
L Precentral *
L Postcentral *
R Occipital Pole 3 *
R Occipital Pole *
R Occipital Pole 2 *
-1
-0.8
-0.6
-0.4
-0.2
0
0.2
0.4
0.6
0.8
1
B. Resting pVMPFC Connectivity
Major Depressive Disorder Patients
Healthy Controls
Anhedonia Partial Correlation Controlling for Age and General Distress
General Distress Partial Correlation Controlling for Age and Anhedonia
